# Removal of the Potassium Chloride Co-Transporter from the Somatodendritic Membrane of Axotomized Motoneurons Is Independent of BDNF/TrkB Signaling But Is Controlled by Neuromuscular Innervation

**DOI:** 10.1523/ENEURO.0172-19.2019

**Published:** 2019-10-15

**Authors:** Erica Tracey Akhter, Ronald W. Griffith, Arthur W. English, Francisco J. Alvarez

**Affiliations:** 1 Departments of Physiology, Emory University, Atlanta, GA 30322; 2 Cell Biology, Emory University, Atlanta, GA 30322

**Keywords:** excitability, GABA, KCC2, nerve injury, neuromuscular junction, regeneration

## Abstract

The potassium-chloride cotransporter (KCC2) maintains the low intracellular chloride found in mature central neurons and controls the strength and direction of GABA/glycine synapses. We found that following axotomy as a consequence of peripheral nerve injuries (PNIs), KCC2 protein is lost throughout the somatodendritic membrane of axotomized spinal cord motoneurons after downregulation of *kcc2* mRNA expression. This large loss likely depolarizes the reversal potential of GABA/glycine synapses, resulting in GABAergic-driven spontaneous activity in spinal motoneurons similar to previous reports in brainstem motoneurons. We hypothesized that the mechanism inducing KCC2 downregulation in spinal motoneurons following peripheral axotomy might be mediated by microglia or motoneuron release of BDNF and TrkB activation as has been reported on spinal cord dorsal horn neurons after nerve injury, motoneurons after spinal cord injury (SCI), and in many other central neurons throughout development or a variety of pathologies. To test this hypothesis, we used genetic approaches to interfere with microglia activation or delete *bdnf* from specifically microglia or motoneurons, as well as pharmacology (ANA-12) and pharmacogenetics (F616A mice) to block TrkB activation. We show that KCC2 dysregulation in axotomized motoneurons is independent of microglia, BDNF, and TrkB. KCC2 is instead dependent on neuromuscular innervation; KCC2 levels are restored only when motoneurons reinnervate muscle. Thus, downregulation of KCC2 occurs specifically while injured motoneurons are regenerating and might be controlled by target-derived signals. GABAergic and glycinergic synapses might therefore depolarize motoneurons disconnected from their targets and contribute to augment motoneuron activity known to promote motor axon regeneration.

## Significance Statement

The neuronal potassium-chloride cotransporter KCC2 is dysregulated after numerous types of neuronal injuries and has been related to neuronal dysfunction, hyperalgesia and spasticity. In this study, we investigated KCC2 regulation on spinal motoneurons with axons injured during peripheral nerve transections. We illustrate that KCC2 loss on axotomized motoneurons occurs at the transcriptional level. This loss differs in time course, completeness and signaling mechanisms from KCC2 dysregulation in other neurons after various pathologies or in motoneurons after spinal cord injury (SCI). In contrast, KCC2 loss on axotomized motoneurons relates to muscle innervation, suggesting a dependence on target-derived signals. We argue that KCC2 loss in axotomized motoneurons may be part of the response that facilitates regeneration of motor axons in the periphery.

## Introduction

Peripheral nerve injury (PNI) affects over 200,000 people annually in the United States and recovery outcomes are poor, including loss of sensorimotor function that can persist for years (Mundie, 1920; Taylor et al., 2008; Höke and Brushart, 2010). A major problem hindering recovery is the slow speed of regeneration of axons in the periphery (1–3 mm/d; Sunderland, 1947; Brushart, 2011). Treatments that accelerate motor axon growth include exercise and electrical stimulation, both dependent on activity and the release of BDNF (Al-Majed et al., 2000a,b; English et al., 2007, 2009; Udina et al., 2011). Counterintuitively, there is also a loss of excitatory synapses on the cell bodies of axotomized neurons while inhibitory synapses are largely retained (Lindå et al., 1992, 2000; Brännström and Kellerth, 1998; Novikov et al., 2000; Alvarez et al., 2011; Rotterman et al., 2019). This change in excitatory/inhibitory balance is usually interpreted as a mechanism to counter hyperexcitability such that motoneurons enter a “quiet” phase, focusing resources in gene and protein expression to promote regeneration (Choi, 1992; Oliveira et al., 2004). Indeed, enhanced preservation of inhibitory synapses in axotomized motoneurons increases the speed of muscle reinnervation and recovery of motor function (Berg et al., 2012).

However, despite the common argument that inhibitory synapse preservation helps keep injured motoneurons quiescent, GABAergic and glycinergic synapses on axotomized motoneurons may not provide effective inhibition. Isoform two of the potassium chloride cotransporter (KCC2) regulates intracellular chloride in most adult neurons; it defines the strength of inhibition (Payne et al., 2003; Kaila et al., 2014; Kahle and Delpire, 2016) and even the direction of membrane polarizations after opening of GABA_A_ and glycine receptor chloride channels. KCC2 expression is downregulated in brainstem motoneurons following peripheral axotomy (Nabekura et al., 2002; Toyoda et al., 2003; Tatetsu et al., 2012; Kim et al., 2018) and results in spontaneous membrane depolarizations dependent on GABA_A_ and NMDA receptor activation that trigger calcium entry through the opening of voltage-gated calcium channels (Toyoda et al., 2003).

KCC2 expression in spinal motoneurons after peripheral axotomy has not been investigated. Most studies on adult spinal motoneuron KCC2 relate to dysregulation after spinal cord injury (SCI), which results in disinhibition and behavioral spasticity (Boulenguez et al., 2010; Murray et al., 2011; Viemari et al., 2011; Bos et al., 2013). Studies investigating spinal KCC2 after PNI, however, have focused on KCC2 dysregulation in dorsal horn neurons associated with mechanisms that promote hyperalgesia (also by disinhibition; Coull et al., 2003, 2005; Ferrini and De Koninck, 2013). In all of these conditions, decreases in KCC2 are considered maladaptive. As such, pharmacological enhancement of KCC2 activity has been a focus for targeted treatments of spasticity and neuropathic pain (Gagnon et al., 2013; Kahle et al., 2014; Sánchez-Brualla et al., 2018). KCC2 loss after PNI or SCI was found to be rapid but incomplete, and was the consequence of altered KCC2 trafficking and/or stability at the membrane (Rivera et al., 2002; Bos et al., 2013; Kahle et al., 2013; Medina et al., 2014). In particular, a microglia-BDNF-TrkB pathway is often highlighted as responsible for downregulation of KCC2 in spinal neurons after PNI (Coull et al., 2005; Ferrini and De Koninck, 2013). Other studies also point to BDNF-TrkB signaling as responsible for regulating KCC2 levels during development and under a variety of disease conditions, acting on both membrane trafficking and gene expression (Lee-Hotta et al., 2019). In contrast to the above studies, spinal motoneurons are directly injured (axotomized) after PNI, and whether KCC2 is also lost and whether it involves similar mechanisms of BDNF and TrkB signaling is unknown. The mechanisms that downregulate KCC2 in brainstem motoneurons after PNI have not been elucidated.

In this study, we show that KCC2 is decreased in spinal motoneurons after PNI. This loss is more extensive than after SCI and occurs throughout the somatodendritic membrane, but proceeds with a relatively slow time course (weeks instead of hours) and after downregulation of KCC2 mRNA. Using genetic approaches and pharmacological manipulations, we also show that the mechanism is independent of microglia activation and BDNF or TrkB signaling. However, we observed an intriguing dependence of KCC2 recovery on neuromuscular junction (NMJ) innervation, indicating that target-dependent factors might be involved in KCC2 regulation. The tight link between KCC2 and regeneration state raises the possibility that the absence of KCC2 from disconnected motoneurons is an adaptive phenomenon related to regeneration mechanisms, an idea we further explore in the discussion.

## Materials and Methods

Animal care, procedures, and euthanasia were performed under prior approval by the Institutional Animal Care and Use Committee of Emory University and in accordance with the NIH Guide for the Care and Use of Laboratory Animals.

### Animals

Several lines of transgenic animals in addition to wild types (WTs) of both sexes were used. Except where noted, mice were obtained from The Jackson Laboratory and maintained on a mixed C57Blk/6J and 6N background. Table 1 lists all transgenic mouse lines used, their JAX codes, and RRID numbers.

**Table 1.** Mice used to derive experimental genotypes

Mouse strain	Stock no.	References
CX3CR1^EGFP^	RRID:IMSR_JAX:005582	Jung et al. (2000)
CX3CR1^creER/+^	RRID:IMSR_JAX:020940	Yona et al. (2013)
CSF1^ff^	Donated Dr. Jean X. Jiang, University of Texas, San Antonio, TX	Harris et al. (2012)
SLICK-A	RRID:IMSR_JAX:007606	Young et al. (2008)
F616A	RRID: IMSR_JAX:022363	Chen et al. (2005)
ChAT^iRES-cre/+^	RRID:IMSR_JAX:006410	Rossi et al. (2011)

To label microglia, we used *Cx3cr1^GFP/+^* heterozygous mice that carry green fluorescent protein (GFP) replacing a single copy of the endogenous fractalkine receptor gene, thereby enabling visualization of microglia (Jung et al., 2000). CX3CR1 is expressed exclusively in microglia in the CNS and subsets of myeloid cells in the periphery (Mizutani et al., 2012). In *Cx3cr1^GFP/GFP^* homozygous mice, both alleles are replaced; these animals lack CX3CR1 expression and display altered microglia function in several diseases (Limatola and Ransohoff, 2014).

To target microglia for cell-specific deletions of BNDF we used tamoxifen-inducible cre, crossing *Cx3cr1^creER/+^* mice with mice carrying floxed *bdnf* alleles (*bdnf^f/f^*). Although CX3CR1 is expressed in peripheral myeloid cells as well as microglia, peripheral myeloid cells have a higher turnover rate (Bennett et al., 2016). Thus, four weeks after tamoxifen treatment only microglia remain *bdnf* genetic knock-outs (KOs), while CX3CR1-expressing myeloid cells have been newly generated from myeloid precursors that lack CX3CR1 expression and have therefore not undergone tamoxifen-induced cre recombination (Goldmann et al., 2013; Tay et al., 2017)

To prevent the microglia reaction after PNI, specifically in the ventral horn of the spinal cord, mice were studied in which the *csf1* gene for colony stimulating factor 1 (CSF1) was knocked out in motoneurons. CSF1 is typically released from injured neurons to activate and recruit microglia (Elmore et al., 2014; Guan et al., 2016). We crossed *Chat^iREScre/+^*with *csf1^f/f^* animals to eliminate CSF1 release from cholinergic neurons, including axotomized motoneurons. This manipulation has been shown to greatly attenuate the ventral horn microglial response to PNI (Rotterman et al., 2019). Mice carrying *csf1^f/f^* alleles were generously donated by Dr. Jean X. Jiang (University of Texas, San Antonio, TX).

We also crossed *Chat^iREScre/+^* animals with *bdnf^f/f^* mice to remove BDNF expression from motoneurons after injury. In this case, the *bdnf* gene was deleted from motoneurons throughout development. To delete *bdnf* more specifically in adult motoneurons, we used tamoxifen inducible single-neuron labeling with inducible CreER-mediated KO (SLICK) mice. Specifically, mice of the SLICK-A line were crossed to *bdnf^f/f^* mice. SLICK-A mice express YFP and tamoxifen-inducible cre in subsets of neurons controlled by the *thy1* promoter (Young et al., 2008). When treated with tamoxifen, cre recombinase is activated in YFP+ neurons and eliminates expression of floxed genes. Thus, after tamoxifen treatment, YFP+ axotomized motoneurons (expressing cre) can be compared with YFP– axotomized motoneurons (not expressing cre) within the same animal (Young et al., 2008; Wilhelm et al., 2012; Zhu et al., 2016).

To investigate the difference between α and γ motoneurons, one animal underwent unilateral sciatic nerve cut and ligation without prior retrograde injections. γ Motoneurons are not typically labeled by retrograde tracers and general markers for motoneurons (ChAT) and neuronal injury (activating transcription factor 3, ATF3) in addition to cell size were used to identify and quantify KCC2 on different populations of injured motoneurons as described below.

### Tamoxifen treatment

When using tamoxifen-inducible cre mice, the drug (0.75 mg/20 g body weight, prepared in 10% ethanol, 90% sunflower oil) was administered via modified gavage once a day for 3 d. Animals were allowed to recover for two weeks, and dosed again for 3 d to ensure complete induction of cre expression. This protocol has been well established as sufficient to induce recombinase activity in SLICK animals (Wilhelm et al., 2012; Zhu et al., 2016). There was a minimum of two weeks between treatment and retrograde tracer injections in SLICK animals. In *Cx3cr1^creER/+^* animals, this interval was extended to four weeks to ensure specificity of gene deletions within only microglia, as described above.

### Retrograde tracer injections

This study focused on one motor pool axotomized after sciatic nerve injuries, the motoneurons innervating the lateral gastrocnemius (LG) muscle. LG Motoneurons were retrogradely labeled either by intramuscular injection of the long-lasting, non-toxic tracer fast blue (FB; Polysciences, Inc) or an adeno-associated virus expressing mCherry (AAV1-mCherry).

FB was injected unilaterally or bilaterally into the LG muscles of adult animals. The animals were anesthetized with isoflurane (4% induction, 2% maintenance) and given preoperative buprenorphine (0.05 mg/kg, i.p.). Once the animals reached a surgical plane of anesthesia, a small skin incision was made to expose the LG muscles, and a 10-μl Hamilton syringe was introduced to inject 2–5 μl of a 1.5% solution into the belly of the muscle. Skin incisions were sutured and the animals were allowed to recover for at least 7 d before nerve surgery to ensure complete retrograde transport.

To visualize the full dendritic arbor of axotomized motoneurons, a small subset of animals were injected with AAV1-mCherry (2 μl of ∼10^9^ IU/m) into the LG muscle at postnatal day 15 with the same anesthesia and analgesia regime as above. Viral injections were performed with glass microelectrodes inserted into the muscle through the skin. These animals were allowed to survive until adulthood (two to three months) before further manipulation. Injections in young animals greatly increase AAV1 infection rates.

### Surgeries

All nerve surgeries were performed under isofluorane anesthesia (4% induction, 2% maintenance) and the animals were given preoperative 0.05 mg/kg buprenorphine (intraperitoneal) to manage possible post-operative pain. The sciatic nerve, which includes the axons of the LG and other hindlimb muscles, was exposed by a mid-thigh skin incision and blunt dissection of the overlying biceps femoris. In animals in the cut/ligated condition, a silk suture was tied tightly around the sciatic nerve and then cut with sharp microscissors ∼2 mm below the ligation. In animals in which the sciatic nerve was cut and repaired, a small rectangle of SILASTIC film (Dow Corning No. 501-1) was placed beneath the nerve where it was secured with fibrin glue (∼5 μl, 2:1:1 thrombin, fibrinogen, fibronectin, MP BioChemicals, LLC catalog #154163, E.C. 3.4.21.5; fibrinogen, Sigma catalog #F3879, E.C. 2325986; fibronectin, Sigma catalog #F1141, E.C. 2891492) before transecting the nerve so that the proximal and distal segments aligned, as has been described elsewhere (English, 2005; Sabatier et al., 2008; Akhter et al., 2019). Sham animals had the sciatic nerve exposed but not transected.

### Tissue collection, processing, and immunocytochemistry

Animals were allowed to survive 3, 7, 14, 21, 28, or 60 days (d) after nerve surgeries, depending on the experiment. They were then overdosed with Euthasol (100 mg/kg) and transcardially perfused with saline-heparin followed by paraformaldehyde (PFA; 4% in 0.1 M phosphate buffer). The spinal cord and injected muscles were harvested and postfixed overnight in 4% PFA before cryoprotection in 30% sucrose for at least 24 h. The spinal cord dura was then removed and the L3–L5 segments isolated. The spinal cords were sectioned in a transverse plane on a freezing sliding microtome at 50-μm thickness and collected free-floating. To improve antibody penetration, the sections were heated for 20 min in 0.01 M sodium citrate with 0.05% Tween (pH 6, maximum temperature 95°C), washed in 0.01 M PBS with 0.3% Triton (PBST), and blocked in normal donkey serum (NDS; 10% in PBS with 1% Triton) for 1 h. The tissue was then incubated, while shaking for two nights at room temperature, with a mixture of appropriate primary antibodies diluted in PBST. The antibodies used, their sources, and RRID numbers are listed in Table 2.

**Table 2.** Primary antibodies used for immunohistochemistry (IHC) and Western blottings (WB)

Antigen	Immunogen	Host/type	Manufacturer	RRID #	dilution	Use
KCC2	N-terminal His-tag fusion protein; pan-rat KCC2; aa 932–1043	Rabbit/ polyclonal	Millipore catalog #07-432,	AB_11213615	1:500	IHC
NeuN	Purified cell nuclei from mouse brain	Mouse/ monoclonal A60 clone	EMD Millipore catalog #MAB377	AB_2298772	1:500	IHC
EGFP	Recombinant GFP 6-his tag	Chicken/ polyclonal	Serotec catalog #obt1644	AB_10000240	1:1000	IHC
Iba1	C terminus of Iba1	Goat/ polyclonal	Novus catalog #NB 100-1028,	AB_521594	1:500	IHC
mCherry	Purified recombinant peptide produced in *E. coli*	Goat/ polyclonal	MyBioSource Catalog #MBS448050,	NA	1:100	IHC
NF-H	Purified NF-H from bovine brain	Chicken/ polyclonal	Aves catalog #NFH	NA	1:500	IHC
VAChT	Recombinant protein; rat VAChT; aa 475–530	Guinea pig/ polyclonal	Synaptic Systems catalog #139105	NA	1:500	IHC
ChAT	Human placental enzyme	Goat/ polyclonal	Millipore catalog #AB144P	AB_2079751	1:100	IHC
ATF3	Recombinant protein corresponding to aa MMLQHPGQVSASEVSASAIVPCLSPPGSLVFEDFANLTPFVKEELRFAINQNKHLCHRMSSALESVTVSDRPLGVSITKAIVAPEEDERKKRRRERNKIAAAKCRNKKKEKTEC	Mouse/ monoclonal	Novus NBP2-34489	NA	1:200	IHC
Phospho-p44/42 MAPK (Erk1/2)	Synthetic phosphopeptide; human p44 MAP kinase; Thr202/Tyr204 residues	Rabbit/ polyclonal	Cell Signaling Technology catalog #9101	AB_331646	1:1000	WB
β-actin	Synthetic peptide; human β-actin; N-terminal region	Rabbit/ polyclonal	Novus catalog #NB600-503	NA	1:5000	WB

Most sections were reacted with primary antibodies against KCC2 and NeuN, and some sections additionally included antibodies to detect microglia (anti-Iba1 or anti-GFP for CX3CR1-GFP amplification in mice carrying this reporter protein). To investigate potential differences between γ and α motoneurons, these sections were reacted with an antibody against ChAT and ATF3 to confirm the injured motoneuron population (Table 2). ATF3 is upregulated in the nucleus of all axotomized neurons (Tsujino et al., 2000). In all cases, the primary antibody against KCC2 was raised against a peptide sequence containing aa 932–1043, which is shared by both KCC2 isoforms (KCC2a and KCC2b). Sections were then washed in PBST and incubated with appropriate fluorescent-coupled species-specific secondary antibodies for 2 h (1:100 in PBST; Table 3). Immunoreactivity (IR) to KCC2 was visualized after incubating with an Alexa Fluor 647-coupled secondary antibody, NeuN-IR with cyanine Cy3-coupled antibody, and Iba1-IR or GFP with FITC-coupled secondary antibodies. LG motoneurons retrogradely labeled with FB were visualized by their fluorescence (excitation 405 nm; emission 420 nm). After washing, the sections were coverslipped with VECTASHIELD (Vector Labs catalog #H-1000) and imaged on an Olympus FLUOVIEW FV1000 Confocal Microscope with 10× or 20× objectives and then at high-magnification using a 60× objective with no digital zoom (NA, 1.35, oil-immersion).

**Table 3.** Secondary antibodies used for immunohistochemistry (IHC) and Western blottings (WB)

Antigen	Manufacturer	RRID#	Use
Alexa Fluor 647 α rabbit	Jackson ImmunoResearch Labs catalog #711-605-152	RRID:AB_2492288	IHC
Cyanine Cy3 (Cy3 α mouse)	Jackson ImmunoResearch Labs catalog #715-165-150	RRID:AB_2340813	IHC
Fluorescein (FITC) α chicken	Jackson ImmunoResearch Labs catalog #703-095-155	RRID:AB_2340356	IHC
Fluorescein (FITC) α goat	Jackson ImmunoResearch Labs catalog #705-095-147	RRID:AB_2340401	IHC
Cyanine Cy3 (Cy3 α goat)	Jackson ImmunoResearch Labs catalog #705-165-147	RRID:AB_2307351	IHC
Cyanine Cy5 (Cy5 α guinea pig)	Jackson ImmunoResearch Labs catalog #706-175-148	RRID:AB_2340462	IHC
HRP-conjugated donkey anti-rabbit	GE Healthcare catalog #GENA934	RRID:AB_2722659	WB

Sections containing AAV1-mCherry motoneurons were reacted with antibodies against KCC2 and IR was detected with Cy5-conjugated secondary antibodies. In these sections, the mCherry signal was amplified by incubating with an antibody against mCherry revealed with Cy3-conjugated secondary antibodies. In addition, these animals were CX3CR1-GFP, and GFP was amplified with anti-GFP and revealed with FITC-conjugated secondary antibodies.

In muscles harvested from sciatic nerve transection-repair animals, cryostat sections were cut at 25-μm thickness and collected onto glass slides. After washing, the slides were incubated for 1 h with 10% NDS. Then they were incubated overnight at room temperature with α-bungarotoxin conjugated to Alexa Fluor 555 (αBgTx-555, Invitrogen catalog #B13422, 1:100) and primary antibodies against neurofilament-H (NF-H) to label the axonal cytoskeleton in thick motor axons and against the VAChT to label the presynaptic motor nerve terminals (Table 2). VAChT-IR was revealed with Cy5-conjugated secondary antibodies and NF-H with FITC-conjugated antibodies (dilution 1:100; Table 3). Sections were mounted with VECTASHIELD and 50 motor endplates per animal were imaged for quantification with an Olympus FLUOVIEW FV1000 Confocal microscope (20×).

### Quantification of KCC2-IR in the cell surface

**Figure 1.**
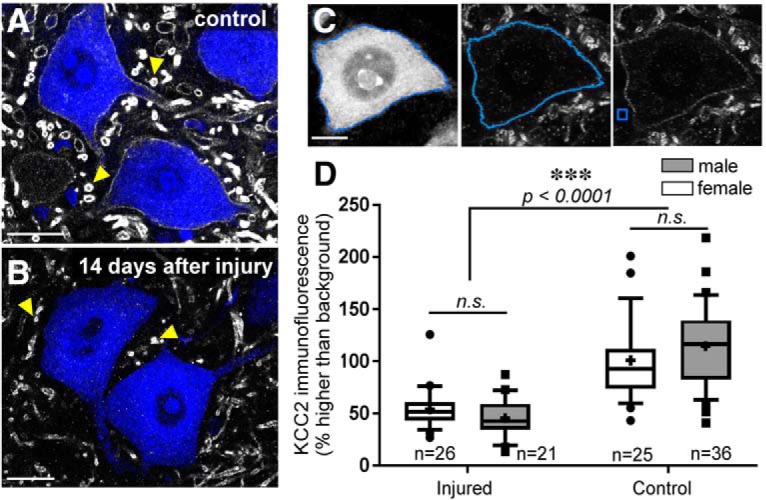
KCC2 is depleted on motoneuron somata 14 d after injury. ***A***, ***B***, LG motoneurons of mice labeled with FB; KCC2-IR in white: sham (***A***) and cut/ligated (***B***). The axotomized motoneurons lose KCC2 protein on the soma membrane, but in some dendrites in the neuropil, KCC2 is preserved (yellow arrows). Scale bar = 25 μm. ***C***, Images of a sham-injured LG motoneuron show the method of KCC2 quantification. Scale bar = 10 μm. Left, Sham-injured LG motoneuron filled with FB. The blue line marks the automatic tracing of the soma. Middle, Image of the KCC2 channel of same motoneuron with the cell membrane line from which fluorescent intensity is measured. Right, KCC2 immunofluorescence with example 9 μm^2^ of area from which background fluorescence was measured. ***D***, Results of quantitative measures of motoneuron soma surface KCC2-IR in male and female mice 14 d after sciatic ligation. No significant sex differences in KCC2-IR were found; only injury state predicted KCC2 levels (Table 4). Box plots represent 25th, median, and 75th percentiles; whiskers = 10th and 90th percentiles; outliers = data points outside whiskers. Crosses = mean; *n* = number of animals. Each animal estimate was obtained from 10 MNs. n.s. = not significant.

Motoneurons in which somatic KCC2 levels were measured were always filled with FB and imaged at 60 × 1. Unless indicated otherwise, 10 FB+ motoneurons ipsilateral to the injury were analyzed. In the case of animals with the contralateral side used as intact control, 10 FB+ motoneurons were also analyzed on the contralateral side. For all motoneurons, a single z-plane (focal depth ∼0.49 μm at 633-nm excitation with refractory index *n* = 1.518 oil immersion 60× objective of NA 1.35) was selected for each FB labeled motoneuron studied. These planes always included a nucleolus and had minimal dendritic departures from the soma. NeuN-IR was used to aid in finding mid-plane optical sections within the z-stack. KCC2 immunofluorescence intensity around the soma of each motoneuron was quantified using FIJI (Fig. 1*C*). The Wand (tracing) Tool (mode: 8-connected; tolerance: 1000) was used to automatically select the edge of the labeled soma containing FB. This tracing was converted to a 0.42-μm wide line superimposed on the neuronal plasma membrane. The average KCC2 Cy5 fluorescent intensity along the line was calculated (gray level, 12 bits). In each section, background measurements were taken from a 9 μm^2^ square region of the neuropil in the same optical plane, adjacent to the motoneurons and lacking labeled dendrites. KCC2 immunofluorescence was corrected against this background level by calculating the percentage higher than background [100 × (membrane intensity average – background intensity average)/average background intensity]. Data points are presented as averages for each animal, each obtained from 10 motoneurons per animal and side of the spinal cord. Different conditions (i.e., injury, sham, time after injury, genotype, spinal cord side) were compared by obtaining averages of *n* = 4–7 animals.

To investigate the differences between α and γ motoneurons, motoneurons in Lamina IX were visualized with ChAT and those axotomized by sciatic transection were identified with ATF3 labeling. Injured motoneurons and those in the same motor pool on the contralateral side were manually traced because ChAT is downregulated after injury and cannot be reliably recognized automatically. Quantification of KCC2-IR was performed as described above. The neurons were designated as γ motoneurons if they had mid-plane cross sectional areas smaller than 475 μm^2^ (criteria published in Shneider et al., 2009) and equivalent numbers of injured and uninjured α and γ motoneurons were quantified (*n* = 16 injured, *n* = 25 uninjured).

### Quantification of KCC2-IR in the cytoplasm

In animals euthanized 3 d after sciatic nerve cut and ligation, KCC2-IR was quantified in the cytoplasm to address whether KCC2 disappearance from the membrane may have been, in part, explained by increased KCC2 internalization or decreased insertion into the membrane. In the same 10 motoneurons quantified for membrane KCC2-IR, samples of KCC2-IR were taken from five 9-μm^2^ rectangles per motoneuron placed on the cytoplasm. These areas were selected in the FB channel to eliminate the potential for bias and they did not include areas of the nucleus or nucleolus. The average cytoplasmic KCC2-IR was calculated for each motoneuron and then corrected for background as described for the membrane measurements.

### Qualitative analyses of KCC2 in motoneuron dendritic arbors

**Figure 2.**
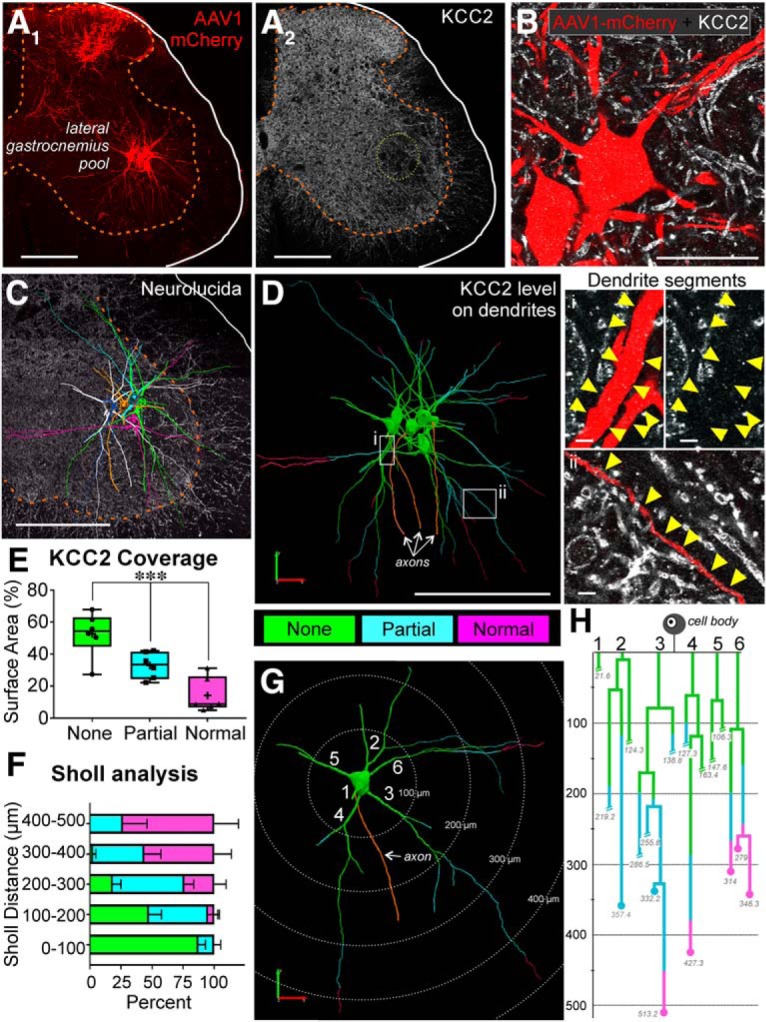
KCC2 protein is lost extensively from axotomized motoneuron dendrites 14 d after sciatic cut/ligation. ***A***, AAV1-mCherry filled LG motoneurons (***A_1_***) and KCC2-IR in the same section (***A_2_***) 14 d following sciatic cut/ligation. Note some afferents are also labeled in the dorsal horn after AAV1 mCherry muscle injections. Scale bar = 200 μm. ***B***, High-magnification confocal image through the soma and proximal dendrites of two mCherry-labeled and axotomized motoneurons (mCherry-red). KCC2 immunofluorescence (white) was not observed around the cell body and the proximal dendrites. Scale bar = 50 μm. ***C***, The full dendritic arbor was imaged at high magnification using confocal image tiling and then the neurons reconstructed in 3D using Neurolucida. Example of six individual motoneurons (color-coded) reconstructed within one section with KCC2-IR in the background. Note the extension of dendrites toward the dorsal horn and also entering the white matter. Scale bar = 200 μm. ***D***, Dendrites were color-coded to indicate complete KCC2 loss (green, “none”), partial KCC2 loss (blue), or normal KCC2 levels (pink), relative to surrounding dendrites in the neuropil. Loss of fluorescence in the proximal dendrites was similar to that on the soma membranes (green and region i). IR to KCC2 was partially reduced (blue and region ii) along dendrites for various distances. However, no significant loss of KCC2 was found on distal dendrites; scale bars (i, ii) = 5 μm. ***E***, Box plots of the percentage of surface area in each category (complete depletion, partial depletion, or no depletion) for all dendrites for which the complete length was traceable (not cut short during sectioning). More than half of the available dendritic surface area was completely depleted (Table 4; ***p < 0.001). Box plots represent 25th, median, and 75th percentiles; whiskers = 10th and 90th percentiles. Crosses = mean; points represent average for individual neurons (*n* = 6, 3.12 ± 0.98 dendrites/animal). ***F***, ***G***, Sholl analysis (100-μm bins) of dendritic length and KCC2 depletion. ***F***, Average (±SD) percentage of dendritic lengths within each bin (*n* = 7 neurons, 5.29 ± 1.1 dendrites per neuron), illustrating that within the first 200 μm, the majority of dendrites have no KCC2 visible, whereas normal KCC2 levels can be observed at the most distal ends of dendrites. ***G***, Isolated single motoneuron from ***C*** separated for Sholl analysis. The majority of KCC2 is completely lost in the first 200 μm of dendrite; at 400 μm, dendritic KCC2 is largely preserved. ***H***, Example dendrogram of the motoneuron in ***G***, color-coded for KCC2 depletion. Dendrite branches ending in circles represent dendrites that were fully contained within the section (quantified in ***E***). Dendrite branches that were cut during sectioning and could not be fully traced are designated with slashes. The majority of dendrites for which the ending could be observed had normal levels of KCC2 present in their terminal regions.

Spinal cord sections containing AAV1-mCherry filled motoneurons and KCC2-IR were imaged at high magnification (60 × 1). At this magnification, the field of view includes only a small region of the whole dendritic arbor in the section. To image the entirety of the dendritic arbor contained within the section we used image tiling of contiguous z-stacks. The images were imported into Neurolucida (v10.0, MBF Bioscience) to fully reconstruct the cell bodies and dendritic arbors contained within the 50-μm-thick section. The dendritic arbors contained within the section were reconstructed in seven motoneurons 14 d after axotomy. Following reconstruction, KCC2-IR labeling in dendritic processes was designated as complete KCC2 depletion (Fig. 2, green), partial depletion (Fig. 2, blue), or normal levels of KCC2 (Fig. 2, pink), in comparison to relative levels of KCC2 in dendrites in the surrounding neuropil. The distances of these dendritic segments to the cell body and their total surface were tabulated, and a Sholl analysis was performed to calculate the proportion of membrane covered with KCC2-IR at different distances from the cell body, using 100-μm bins.

### ANA-12 treatment

A subset of WT animals was treated continuously from injury to euthanasia with ANA-12, a competitive TrkB antagonist, or vehicle. ANA-12 was prepared in DMSO (30%), PEG300 (30%), and sterile saline (40%) at 2 mg/ml, and delivered (0.5 μl/h) intraperitoneally using an ALZET-2002 mini-osmotic pump. This rate was chosen to deliver the same concentration of ANA-12 per 12 h as when administered twice daily with intraperitoneal injections (Ambrogini et al., 2013; Chen et al., 2015). The use of osmotic pumps allows maintenance of a more consistent concentration of the drug throughout the treatment period. Pumps were filled with ANA-12 or vehicle and primed in sterile saline overnight at 37°C. Pumps were implanted in the animals at the time of nerve cut/ligation (ANA-12, *n* = 6; vehicle, *n* = 4) or sham (ANA-12, *n* = 4). They were brought from warm saline and immediately inserted into the peritoneal cavity, being careful not to disturb internal organs. The wound was closed in layers and animals were monitored daily to insure pump stability. Before perfusion 14 d later, pumps were removed, weighed, and evacuated with a needle to ensure the drug was properly delivered. All pumps had expelled the expected volume of drug.

A subset of animals (*n* = 2 per group: sham-vehicle, sham-ANA-12, injury-vehicle, injury-ANA-12) was used to confirm the effectiveness of the ANA-12 treatment. Western blots were used to analyze phosphorylation of ERKs (pERKs), a downstream effector of TrkB activation known to increase in the spinal cord after nerve injury (Wang et al., 2004). Animals were treated with ANA-12 or vehicle (as above) and underwent bilateral sham or sciatic nerve cut/ligations. Seven days postinjury, the animals were euthanized and rapidly perfused with ice-cold saline, and the lumbar spinal cords were extracted and flash frozen. The tissue was homogenized in phosphatase-inhibitory lysis buffer (10 mM HEPES, pH 7.4; 50 mM NaF; 50 mM NaCl; 5 mM EDTA; 5 mM EGTA; 0.1% Triton X-100; 1 mM Na_3_VO_4_; 10 μM leupeptin; and 1 mM phenylmethylsulfonyl fluoride, 25 kIU/ml aprotinin) and centrifuged (13,000 × *g* at 4°C) to separate lysate. The pellet was discarded and cell lysates preserved at –80°C until the samples were assayed for protein concentration and processed for PAGE. Protein concentration was determined with the Bio-Rad DC protein assay (catalog #5000116) at 750 nm. Protein standard dilutions were prepared in buffer containing the protease inhibitors (see above). Forty micrograms of protein from each sample was loaded onto Bio-Rad 10% polyacrylamide precast gels (50-μl wells, catalog #456-0834) and Bio-Rad Kaleidescope protein molecular weight markers (catalog #161-0375) were loaded into at least one well on each gel. After electrophoresis, Western blotting was conducted overnight at 4°C onto PVDF membranes (Bio-Rad catalog #162-0177) using 150-mA constant current. Primary rabbit antibodies against phospho-p44/42 MAPK (pMAPK) or pERK (Thr202/Thr204) and β-actin were used (Table 2). The secondary antibody in all cases was HRP-conjugated donkey anti-rabbit, and protein immunostaining was revealed using chemiluminescence (Clarity ECL Western substrate, Bio-Rad catalog #170-5061). β-Actin was used as a loading control. The membrane was stripped using Re-Blot Plus, Strong Solution (Millipore catalog #2504) before re-probing with anti β-actin.

### Pharmacogenetic TrkB inactivation

F616A mice carry a mutation in the TrkB receptor that makes it sensitive to specific inhibition by the small molecule inhibitor 1-(1,1-dimethylethyl)-3-(1-naphthalenylmethyl)-1H-pyrazolo[3,4-d]pyrimidin-4-amine, PP1 analog (1NMPP1). We administered 1NMPP1 following protocols well established in the literature (Chen et al., 2005). Briefly, 3 d before nerve injury, F616A animals were switched from normal drinking water to drinking water with 1NMPP1 (5 μM, 0.01% DMSO) or vehicle. Animals were monitored twice daily to ensure proper hydration and adequate access to the water.

### KCC2 mRNA detection using RNA-Scope

Standard surgical and euthanasia procedures were performed as described above with the exception that perfusions were performed with 4% PFA in 1× PBS. For RNA-Scope labeling, we prepared animals with sciatic nerve injury ligations. Animals were euthanized 3, 7, and 14 d after nerve surgeries (*n* = 3 animals per group). Following perfusion, spinal cords were harvested and post-fixed overnight at 4°C, followed by stepwise cryoprotection (10% sucrose in 1× PBS overnight, 20% sucrose in 1× PBS overnight, 30% sucrose in 1× PBS overnight) before sectioning (16 μm) on a cryostat. Sections were collected on RNAase free slides. Samples were then sent to Advanced Cell Diagnostics (ACD Bio) on dry ice and processed for RNA-Scope KCC2 mRNA *in situ* hybridization using a probe directed toward the 501- to 1717-bp region of the KCC2 mRNA (Mm-SLC12A5 ACD catalog #311901). This probe detects mRNA for both KCC2a and KCC2b isoforms and consists of 20 Z probe pairs. We used the ubiquitously expressed peptidylprolyl isomerase B (cyclophilin B; ACD catalog #313911) as positive control to confirm mRNA preservation, and dihydrodipicolinate reductase (dapB), a gene from Bacillus subtilis (ACD catalog #310043), as negative control to assess background labeling. To improve tissue adherence, sections were baked at 60°C for 45 min and then further postfixed on the slide (4% PFA in 1× PBS) for 90 min at room temperature before serial dehydration (5 min each; 50% EtOH, 70% EtOH, 100% EtOH, 100% EtOH) and air-drying. Standard RNA-Scope procedures (Wang et al., 2012) were used and the KCC2 mRNA binding detected with RNA-Scope 2.5 HD Reagent Kit-BROWN (catalog #322300). The sections were counterstained with standard Nissl stain (hematoxylin).

### RNA-Scope KCC2 mRNA image analysis

**Figure 3.**
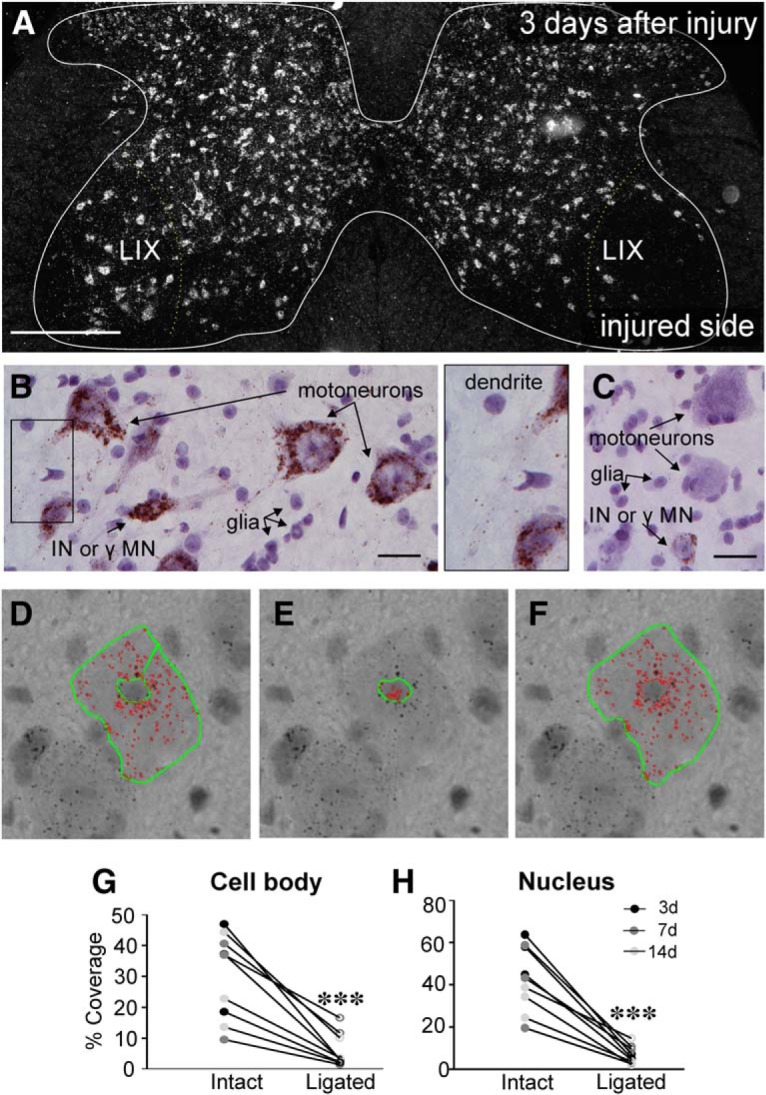
KCC2 mRNA is lost by 3 d after peripheral axotomy. ***A***, Darkfield image of a lumbar 4 section 3 d after unilateral sciatic nerve cut/ligation, processed using RNA-Scope. KCC2 mRNA visualized in white. Scale bar = 200 μm. Very little KCC2 mRNA is found in the area around the injured motor pool (right) in Lamina IX (LIX), relative to the contralateral intact motor pools. ***B***, ***C***, Brightfield images of motoneurons on the intact (***B***) or cut/ligated (***C***) sides of the spinal cord 3 d after PNI. Injured motoneurons lose KCC2 mRNA, whereas motoneurons contralateral to injury and interneurons (INs) or γ motoneurons (γMNs) on both sides of the spinal cord maintain KCC2 mRNA. Nuclei from glia are also visible and are more densely concentrated around axotomized neurons. KCC2 mRNA can also be seen extending into dendritic processes of intact motoneurons (rectangle in ***B***, inset). Scale bar = 25 μm. ***D–F***, RNA-Scope quantification procedure. Thresholding was used to highlight KCC2 mRNA (red). Nissl staining caused differences in background intensity, so difference thresholds were required for cytoplasm and nuclear quantification. Thus, the outlines of the motoneuron somata were traced (green) with the nucleus excluded to get the area of KCC2 mRNA within the cytoplasm (***D***). Nuclei (***E***) were traced and threshold separately. The total area, including cytoplasm and nucleus (***F***), was also traced for comparison of total area to KCC2 mRNA area. ***G***, ***H***, Percentage KCC2 coverage of soma (***G***) and nucleus (***H***). Animals were euthanized 3, 7, or 14 d (*n* = 3/group) after unilateral sciatic nerve cut/ligation, and sciatic motoneurons were quantified as described above. There were no differences between levels of KCC2 between time points; only injury state predicted KCC2 levels (Table 4). Thus, data from all time points were pooled and paired *t* tests were performed comparing motoneurons ipsilateral and contralateral to injury (Table 4; ***p ≤ 0.001).

Dark- and bright-field images were taken on an Olympus BX60 microscope with Spotcam RX3 camera at 10× and 40 × 2 magnification, respectively. Images were imported into ImagePro ver 7.0 (Media Cybernetics), and thresholds for KCC2 mRNA signal were determined to segment the KCC2 mRNA labeling from the image. The same thresholds were used for all sections, but different thresholds were used for the cytoplasm and nucleus as the Nissl stain had a different gray level in nuclei and most KCC2 mRNA signal was usually concentrated on top of the nucleolus. The total area of cytoplasmic or nuclear labeling was calculated (Fig. 3*D–F*) and data presented as average percentage coverage of KCC2 mRNA labeling in each cell compartment. We noted interanimal variability in the strength of the KCC2 mRNA signals. For this reason, we always compared labeling intensities to the contralateral control uninjured side.

### Statistical analysis

Statistical tests were performed as described in the results and powered to power > 0.8 for α < 0.05 (see statistical tables; Tables 4–6). Data were evaluated with *t* tests or one-way or two-way ANOVA as appropriate. Bonferroni *post hoc* multiple comparisons tests were performed against controls (WT or transgene negative control animals) unless otherwise described. Finally, RNA-Scope quantification was compared using paired *t* tests between spinal cord sides ipsilateral and contralateral to the injury. Power was calculated using SigmaPlot (Systat Software, Inc.,12.3) and all other statistics were performed with PRISM (GraphPad, 7.03).

## Results

### KCC2 is lost from the somatic and proximal dendrite membrane of motoneurons axotomized after nerve injury

Using antibodies raised against a rat KCC2 sequence (aa 932–1043) shared by both KCC2 isoforms (KCC2a and KCC2b), we found a loss of KCC2-IR on the sciatic motor pools ipsilateral to the injury after unilateral sciatic nerve transections (Fig. 1). Fourteen days after sciatic nerve transection, KCC2-IR was strongly reduced or absent from the plasma membranes of cell bodies and proximal dendrites of LG motoneurons labeled with FB (Fig. 1*A*,*B*). Using an unbiased automatic detection of the cell body plasma membrane based on FB fluorescence (see Materials and Methods; Fig. 1*C*), we measured KCC2-IR along the membrane. This measurement was then normalized to background fluorescence estimated in the same optical plane. KCC2-IR levels are expressed as a percentage of fluorescence intensity above background (0% = background). Ten motoneurons were studied in each animal, unless otherwise indicated. Thus, *n* always refers to the number of animals analyzed.

In ∼90% of the motoneurons analyzed in sham or naïve animals, levels of KCC2 fluorescence averaged between 75% and 150% above background (Fig. 1*D*). After injury, average KCC2 immunofluorescence estimates dropped to around 50% higher than background, with more than half of the estimates below this level (Fig. 1*D*). At this level of measured KCC2 immunofluorescence above background, no KCC2 immunofluorescence was visible along the plasma membrane. This level above background might be due to an offset gray level imposed by the cytoplasm that is always slightly more fluorescent than neuropil background. Similar numbers of male and female mice underwent axotomy, and at 14 d after injury a significant depletion was found in injured animals, but no sex differences were detected in either intact or injured motoneurons (Fig. 1*D*). A two-away ANOVA for condition (injured vs non-injured) and sex showed significant differences according to condition, but not sex, or the interaction of injury and sex (Table 4). Thus, males and females were pooled together in all experiments described below.

**Table 4.** Details of statistics for experiments describing the phenomenon of KCC2 loss on spinal motoneurons following axotomy

Location	Data reference	Data structure	Type of test	Power and statistical significance
A	1D	Non-normal distribution, unequal variance	Two-way ANOVA Injury; *F*_(1,103)_ = 76.340; *p* < 0.001 Sex; *F*_(1,103)_ = 0.206; *p* = 0.683 Interaction; *F*_(1,103)_ = 3.155; *p* = 0.086	Power = 1.00 Power = 0.05 Power = 0.29
B	2E None vs partial None vs normal Partial vs normal	Normal distribution, unequal variance	One-way ANOVA * F*_(2,17)_ = 17.52; * p* < 0.001 *Post hoc* Bonferroni *Post hoc* Bonferroni *Post hoc* Bonferroni	Power = 0.999 *p* = 0.0218 *p* < 0.0001 *p* = 0.0285
C	3H Intact vs ligated	Normal distribution, unequal variance	Two-way ANOVA Side; *F*_(1,17)_ = 58.314; *p* < 0.001 Day; *F*_(2,17)_ = 1.811; *p* = 0.205 Interaction; *F*_(2,17)_ = 2.308; *p* = 0.142 One-tailed paired *t* test	Power = 1.0 Power = 0.153 Power = 0.223 *t* = 6.07, *p* = 0.0002
D	3G Intact vs ligated	Normal distribution, unequal variance	Two-way ANOVA Side; *F*_(1,17)_ = 58.314; *p* < 0.001 Day; *F*_(2,17)_ = 0.132; *p* = 0.878 Interaction; *F*_(2,17)_ = 0.463; *p* = 0.640 One-tailed paired *t* test	Power = 0.981 Power = 0.05 Power = 0.05 *t* = 7.591, *p* < 0.0001
E	Sham vs ligated		One-tailed *t* test	Power = 0.95 *t* = 0.418 *p* = 0.6893
F	**5D** **α intact vs** α injured γ intact γ injured **α injured vs** γ Intact γ injured **γ intact vs** γ injured	Non-normal distribution, unequal variance	Two-way ANOVA Injury; *F*_(1,82)_ = 0.0003; *p* < 0.001 Type; *F*_(1,82)_ = 17.0; *p* = 0.986 Interaction; *F*_(1,82)_ = 4.1; *p* = 0.046 *Post hoc* Bonferroni *Post hoc* Bonferroni *Post hoc* Bonferroni *Post hoc* Bonferroni *Post hoc* Bonferroni *Post hoc* Bonferroni	Power = 0.987 Power = 0.05 Power = 0.398 *p* = 0.0002 *p* = 0.59 *p* = 0.307 *p* = 0.246 *p* > 0.999 *p* = 0.8565
G	4B **Intact vs** 3D (cut/ligated) 14D (cut/ligated) 21D (cut/ligated) 21D (cut/repair) 28D (cut/ligated) 28D (cut/repair) 60D (cut/ligated) 60D (cut/repair)	Normal distribution	One-way ANOVA *F*_(8,36)_ = 4.194; *p* = 0.002 *Post hoc* Bonferroni *Post hoc* Bonferroni *Post hoc* Bonferroni *Post hoc* Bonferroni *Post hoc* Bonferroni *Post hoc* Bonferroni *Post hoc* Bonferroni *Post hoc* Bonferroni	Power = 0.913 *p* > 0.999 *p* = 0.026 *p* = 0.003 *p* = 0.128 *p* = 0.032 *p* = 0.017 *p* = 0.041 *p* = 1.00
H	4F	Normal distribution	Linear regression *F*_(1,12)_ = 15.942; *p* = 0.002	Power = 0.896

Boldface indicates the group against all other comparisons are made in multiple comparisons tests.

Despite the robust loss of KCC2 on the soma, KCC2-IR can be observed on some small dendrite segments within the neuropil around the cell bodies (Fig. 1*A*,*B*, yellow arrowheads), although at a lower density compared to controls. It is possible they are distal segments of axotomized sciatic motoneurons or that they originate from nearby uninjured interneurons or non-sciatic projecting motoneurons. We were concerned that if KCC2 was preserved on dendrites it could act as a siphon to extrude chloride from somatic and proximal dendrite compartments, minimizing potential chloride accumulation and affecting interpretation of the data with respect to responses to GABA/glycine. Thus, we performed experiments to analyze KCC2 coverage throughout the dendrite plasma membrane 14 d after nerve injury. FB only fills the soma and most proximal dendrites; therefore, to visualize the full dendritic arbor, P15 animals were injected with AAV1-mCherry into the LG. AAV1-mCherry fills the dendritic arbor fully and mCherry expression is preserved until adulthood without impacting motoneuron function (Fig. 2*A*,*B*). These animals underwent sciatic cut/ligation in adulthood, and we analyzed seven motoneurons 14 d after injury. Three-dimensional reconstructions of motoneuron dendritic arbors were obtained using Neurolucida (Fig. 2*C*,*D*). In the proximal dendrites, KCC2 was depleted to a similar extent as in the soma. However, we observed a gradient in which more KCC2-IR could be observed on the dendrites at progressively greater distances from the soma, and on the most distal dendrites KCC2-IR was fully preserved. The extent of KCC2 depletion varied from neuron to neuron and dendrite to dendrite. Overall, 52.6 ± 13.9% (± SD) of the total dendritic surface area had no KCC2-IR, 33.0 ± 8.3% showed a partial preservation of KCC2-IR, and 14.3 ± 10.8% of dendrites surface showed normal KCC2-IR 14 d after injury (Fig. 2*E*). The differences in total surface area with no, partial, or normal KCC2-IR were statistically significant (Table 4). Using Sholl analysis, we found that the areas with full preservation of KCC2 were always distal and regions with total depletion were always proximal (Fig. 2*F*). In dendritic segments located in the first 100 μm of Sholl distance from the cell body, 87.3 ± 14.9% of their surface was depleted of KCC2, and at 100- to 200-μm distance 47.4 ± 26.8% of the surface was totally depleted. However, in dendritic segments above 200 μm of Sholl distance, partial or total preservation of KCC2-IR was commonly observed (Fig. 2*F–H*). In analyses of individual dendrites (Fig. 2*G*,*H*) complete preservation of KCC2-IR was found in the terminal dendritic regions (Fig. 2, dot marks in dendrograms). Not all dendrites were fully contained within the section (hatch marks in dendrograms) and these were not included in the Sholl analysis; mCherry-labeled dendrites ending within the top and bottom surfaces of the section typically display a few ending varicosities. KCC2 preservation in the most distal dendrites was independent of whether the dendrites ended in the gray or white matter (Fig. 2*C*). The impact of KCC2 retention in these distal regions on overall chloride levels might be minimal since motoneuron dendrites at these distances are of very small caliber and contribute little to the total surface; thus, the overall percentage of dendrite surface with normal KCC2 density is quite small (Fig. 2*E*). On the other hand, the impact of partial retention of KCC2 density in mid-distal dendrite regions is yet unknown (see Discussion).

### KCC2 regulation on axotomized motoneurons occurs at the transcriptional level

Disappearance of KCC2 protein from the membrane of axotomized motoneurons could occur because of membrane protein turnover and degradation, slower KCC2 protein trafficking to the membrane, or reduced gene expression. In dorsal horn interneurons and spinal motoneurons after SCI, KCC2 downregulation has been reported to occur rapidly and through post-translational KCC2 phosphorylation mechanisms likely affecting trafficking and membrane stabilization of KCC2 (Coull et al., 2005; Boulenguez et al., 2010; Bos et al., 2013; Ferrini and De Koninck, 2013). However, previous studies in axotomized brainstem cranial motoneurons reported a strong downregulation of KCC2 mRNA after PNI. This results in slower removal of KCC2 from the membrane, but the loss is more profound and longer lasting (Toyoda et al., 2003).

To investigate whether the loss of KCC2 after PNI is the result of reduced gene expression, we measured *kcc2* mRNAs using a diaminobenzidine (DAB)-based RNA-Scope method and a probe that recognizes a 501- to 1717-bp region shared by both *kcc2* isoforms (Fig. 3). In dark-field microscopy we observed complete depletion of *kcc2* mRNA in Lamina IX motoneurons in the region corresponding to the sciatic motor pool ipsilateral to the lesion and little changes in other spinal cord laminae or in Lamina IX motoneurons on the intact contralateral side (Fig. 3*A*). At high magnification, there was robust and consistent *kcc2* mRNA signal in the cell bodies and nucleus of all uninjured spinal cord Nissl-stained neurons (Fig. 3*B*). Within the nucleus of these cells, there was frequently a large spot of reaction product localized on the nucleolar region. In motoneurons (recognized by their large size in Lamina IX), we also observed *kcc2* mRNA reaction product entering proximal dendrites (Fig. 3*B*). Both cytoplasmic and nuclear labeling were strongly decreased in axotomized motoneurons (Fig. 3*C*). We quantified RNA-Scope reaction products by measuring the area covered by reaction particles within both the cell body cytoplasm and nucleus of motoneurons in Lamina IX sciatic motor pools. Different thresholds were used in each cellular compartment (see Materials and Methods) because of differences in intensity of the reaction product and the different gray levels of the Nissl-stained nucleus versus cytoplasm (Fig. 3*D–F*). Since some interanimal variability was observed in the strength of the RNA-Scope reaction, we compared motoneurons ipsilateral and contralateral to the injury within animals. By 3 d after injury, the extent of coverage by *kcc2* mRNA reaction product was reduced by 90.7 ± 1.5% (SE) in the cytoplasm and 89.8 ± 0.6% in the nucleus. These depletions were consistently maintained through 14 d after injury (81.7 ± 2.3% and 80.1 ± 9.4% depletions in cytoplasm and nucleus, respectively). There were no significant differences in the percentage depletion of the *kcc2* mRNA signal in cytoplasm or nucleus 3, 7, and 14 d after injury (Table 4), thus data from all three time points were pooled together. Paired *t* tests showed highly significant reductions in *kcc2* mRNA signal in both cytoplasm and nucleus.

**Figure 4.**
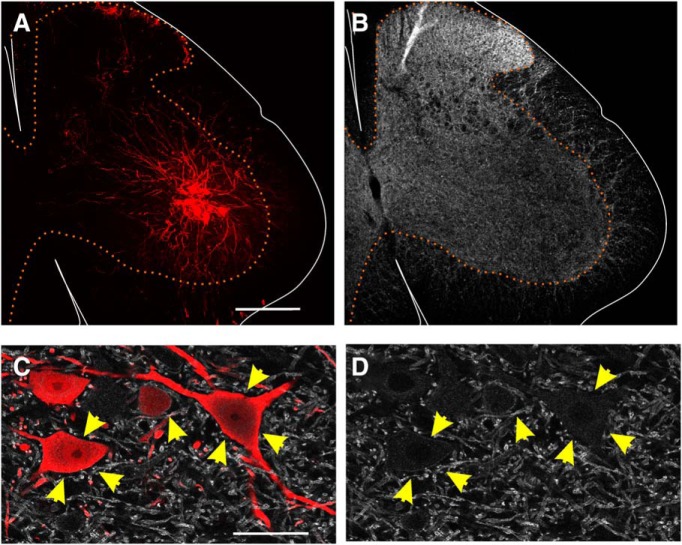
KCC2 protein is not lost on motoneuron somata or dendrites 3 d after sciatic nerve cut/ligation. ***A***, ***B***, AAV1-mCherry filled LG motoneurons (***A***) and KCC2-IR (***B***) in the same section 3 d following sciatic nerve cut/ligation. Scale bar = 200 μm ***C***, ***D***, Representative high-magnification (60× 1) images of the motoneurons (red) above and the KCC2-IR (white) on their somata and dendrites (yellow arrows). There is minimal loss of KCC2-IR on both the soma and dendritic processes. No loss is visible on mid to distal dendrites. Scale bar = 50 μm.

Because KCC2 mRNA was depleted by 3 d after injury, and our time course of KCC2 protein depletion showed a trend toward KCC2 loss on the soma at this time point, we further investigated this time point by examining KCC2 distribution on AAV1-mCherry labeled motoneuron somata and dendrites of animals 3 d after sciatic nerve cut and ligation. Qualitatively, these motoneurons showed no large decreases in KCC2-IR on the soma or throughout the dendrites, further confirming that the mRNA disappears before differences in protein can be observed (Fig. 4). This leads to suggesting that KCC2 loss is due to depletions in *kcc2* transcription and turn-over of membrane KCC2 without replacement by new protein. The possibility that concurrent increased internalization of KCC2 protein is also contributing to the loss observed was tested by quantifying KCC2-IR in the cytoplasm of motoneurons 3 d postinjury. We found no significant differences (*t* test; Table 4) between cytoplasmic KCC2-IR in motoneurons of sham animals (45 ± 7.9% higher than background) and animals in which the sciatic nerve was cut and ligated 3 d prior (50 ± 23.5%). While this finding suggests that the initial depletion of KCC2 is not due to increased internalization of KCC2, it is still possible that any internalized protein is quickly degraded and undetectable with immunocytochemistry. The rate of KCC2 recycling in intact or injured motoneurons is unknown but the rate of KCC2 loss seem quite slow for any significant contributions from rapid enhanced internalization.

**Figure 5.**
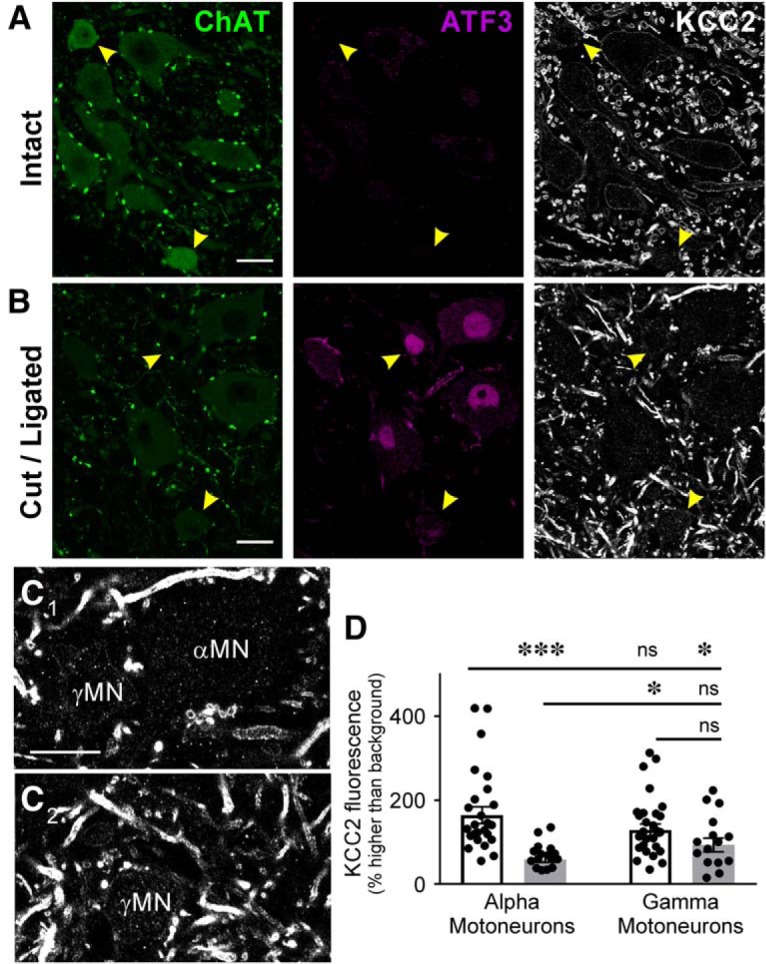
KCC2 protein is not lost as dramatically on γ motoneurons (γMNs) as αMNs. ***A***, ***B***, Confocal images of the ventral horn containing sciatic injured MNs labeled with antibodies against ChAT (green) and ATF3 (red) to mark axotomized MNs, and KCC2 (white) 14 d after sciatic nerve cut/ligation. Uninjured MNs in the side contralateral to the injury (top) lack ATF3 and have similar levels of KCC2-IR in α (large) and γ (<475 μm^2^, yellow arrowheads) MNs. After injury (***B***) KCC2-IR consistently disappears from αMNs, but is more variably depleted in γMNs (yellow arrowheads). ***C***, High magnification of αMNs and γMNs marked in ***B***. γMNs has some KCC2 preserved on the somatic membrane. Scale bars = 20 μm. ***D***, Quantification of KCC2-IR on the membrane of individual MNs (single points) ipsilateral and contralateral to injury. αMNs consistently deplete KCC2 from the somatic membrane after injury, and while there are no statistically different differences in KCC2-IR of intact MNs ipsilateral and contralateral to injury, the difference between injured and uninjured γMNs is not significantly different (Table 4). γMNs do not exhibit KCC2 loss as consistently as αMNs at this time point. ***p < 0.001; *p < 0.05; n.s. = not significant.

RNA-Scope preparations revealed that *kcc2* mRNA was preserved in some small neurons near the large motoneurons we quantified. These could be interneurons or small γ motoneurons. It is impossible to definitively distinguish these different types of neurons in our Nissl-stained RNA-Scope preparations, so we investigated potential differences between γ and α motoneuron KCC2 expression and dynamics by labeling all motoneurons with ChAT antibodies and distinguishing each type based on cell size. γ Motoneurons were not reliably filled by retrograde tracers, so overlapping ChAT and ATF3-IR was used to determine the injured motoneurons. As reported elsewhere, injured motoneurons downregulated ChAT (Ichimiya et al., 2013) and injured motoneuron nuclei were clearly visible by ATF3 labeling (Fig. 5*A*,*B*). Small motoneurons (<475-μm^2^ cross sectional area) were designated as γs, as previously defined (Shneider et al., 2009). KCC2-IR was quantified on the somatic membrane, and although no significant differences between intact α and γ motoneurons (*n* = 25 per group) were detected there was a trend toward lower KCC2-IR in γs. Injured γ and α motoneurons showed significant lower KCC2-IR relative to intact α motoneurons (Table 4). However, KCC2 in γ motoneurons was more variable and many showed KCC2 at normal levels (Fig. 5*C*). This variability and the trend toward diminished KCC2 in γs resulted in no significant differences between intact and uninjured γ motoneurons. Thus, it is possible that γ motoneurons lose KCC2 with different time-courses or not at all, or that there are differences according to the type of γ motoneuron (dynamic vs static). We focused all our analyses on the larger population of α motoneurons that reliably lose KCC2 by 14 d. The interesting possibility of significant differences between γ and α motoneuron KCC2 expression and reactions to injury will need to be investigated in future work.

### Time course of KCC2 protein loss and recovery and its relation with microglial reactivity and muscle innervation

To investigate the time course of KCC2 protein downregulation on axotomized motoneurons and a possible relationship with activation of ventral horn microglia around injured motoneurons, we performed nerve injuries in male and female *Cx3cr1^GFP/+^* heterozygous mice in which microglia express GFP. To analyze the effect of regeneration and muscle innervation in KCC2 expression, we either prevented regeneration by cutting and ligating the sciatic nerve (tying the proximal stump with silk) or enabled regeneration by cutting and repairing the nerve (end-to-end anastomosis of the proximal and distal nerve stumps using fibrin glue). Analyses of KCC2 immunofluorescence were performed in cohorts of animals either without a nerve injury (intact) or 3, 14, 21, 28, or 60 d after injury (*n* = 4 or 5 per group). All analyses were performed on the cell bodies of 10 MNs per animal (as in Fig. 1). Animals with sciatic nerve repair were only analyzed at 21, 28, and 60 d for comparison with cut/ligated animals, as this is when differences in regeneration and muscle reinnervation occur between injury models.

**Figure 6.**
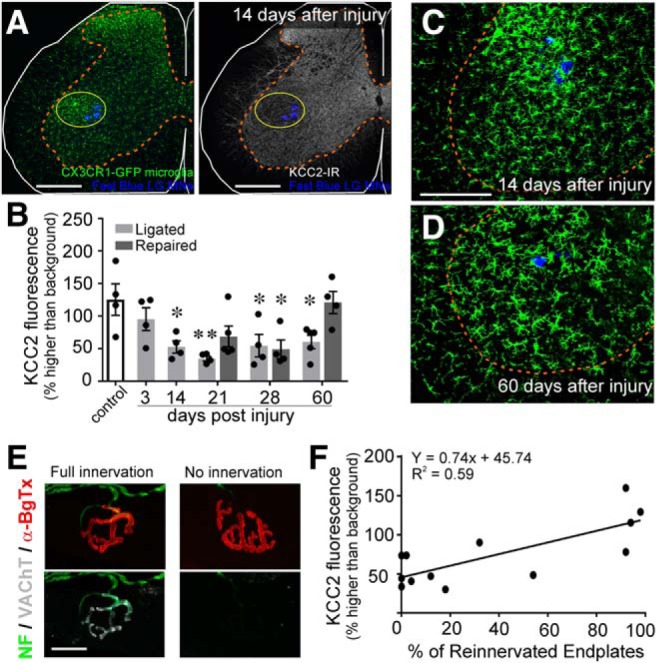
The disappearance of KCC2 coincides with microglial onset but KCC2 restoration depends on muscle reinnervation. CX3CR1^EGFP/+^ animals underwent unilateral sciatic nerve injury and were allowed to survive for various time points. ***A***, Representative images of microglial (green) and KCC2 (white) IR 14 d after sciatic nerve cut/ligation, at the peak of the microglial response. Scale bars = 200 μm. Regions of KCC2 depletion overlap with the area of microglial reactivity. ***B***, Time course of KCC2 downregulation and recovery in CX3CR1^EGFP/+^ animals. KCC2 protein levels begin decreasing by 3 d and are significantly reduced by two weeks following injury but are restored to control levels by 60 d in animals in which regeneration was allowed (repaired; Table 4; *p ≤ 0.05, **p ≤ 0.01). Error bars = SEM. ***C***, ***D***, Microglial reaction (green) around injured LG motoneurons (blue) 14 d (***C***) or 60 d (***D***) after sciatic nerve injury in cut/ligated animals. KCC2-IR loss appears to coincide with the onset of microgliosis but persists after the response has attenuated. ***E***, Representative motor endplates in the LG muscle following sciatic nerve cut/repair. Acetylcholine receptors were labeled with α-bungarotoxin (red) and motor axons were identified by VAChT (white) and NF-H (green). Scale bars = 5 μm. Endplates with overlapping acetylcholine receptors and VAChT were designated as reinnervated. ***F***, Correlation between KCC2-IR and endplate reinnervation of individual animals (circles). Animals with higher levels of reinnervation of the LG have higher levels of KCC2 fluorescence on LG motoneurons (linear regression; Table 4).

At low magnification, KCC2-IR was diminished in the Lamina IX region containing injured sciatic motoneurons (including the FB retrogradely labeled LG motor pool). This area closely overlapped with the ventral horn region showing a strong microglial reaction (Fig. 6*A*). Based on KCC2 quantification on the cell body membrane of FB-labeled LG motoneurons, significant differences were found between control and axotomized motoneurons at different times after injury with regeneration allowed or prevented (Table 4). Using *post hoc* comparisons (Bonferroni-corrected *t* tests), we found that membrane KCC2-IR was significantly greater in control motoneurons than almost all other groups. Notably, KCC2 levels on the somatic membrane of repaired motoneurons 60 d after injury were similar to controls in average and variance (control 125.4 ± 48.4% above background vs 120.9 ± 33.9% in regenerated motoneurons 60 d after injury).

Similar regulation of KCC2 after axotomy was observed in WT animals and *Cx3cr1^GFP/+^*heterozygous animals (see below) as well as *Cx3cr1^GFP/GFP^* animals. We observed no changes in either microgliosis or KCC2 downregulation in *Cx3cr1^GFP/GFP^* animals, where both alleles of the fractalkine receptor are replaced by GFP and thus are *Cx3cr1* KOs (data not shown). KCC2 regulation on the cell bodies of axotomized motoneurons is thus independent of CX3CR1 function. Moreover, microgliosis in the ventral horn is largely resolved by 60 d after injury even when regeneration is not allowed (cut and ligated sciatic nerves; Fig. 6*C*,*D*), without recovery of KCC2.

One interpretation of the above results is that KCC2 restoration on the membranes of motoneurons after PNI occurs only following regeneration of their axons. To investigate whether KCC2 restoration was correlated with successful reinnervation of NMJs, LG muscles from repaired animals were collected and examined for reinnervation of vacated motor endplates by motor axons and presynaptic motor nerve terminals (identified with α-bungarotoxin and IR against NF-H and the VAChT, respectively; Fig. 6*E*). By 60 d after sciatic nerve transection and repair, >95% of motor endplates had been re-occupied by motor nerve terminals (Fig. 6*F*). At earlier time points there was partial reinnervation and more variability from animal to animal. This variability allowed us to correlate NMJ re-innervation levels with KCC2 recovery. We found a statistically significant correlation between the percentage of reinnervated motor endplates within the LG muscle and the average level of KCC2 recovery on the membrane of LG motoneurons (Table 4; Fig. 6*F*).

### KCC2 depletion is independent of microglia.

**Figure 7.**
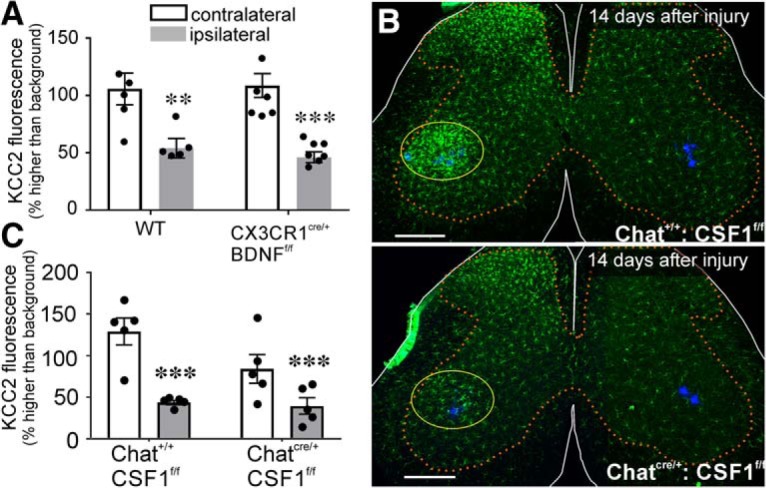
KCC2 depletion occurs independent of microgliosis. ***A***, Relative KCC2 depletion in WT and CX3CR1 BDNF KO animals 14 d after cut/ligation. Genotype had no effect on KCC2 levels in motoneurons contralateral or ipsilateral to injury (Table 5). Removing BDNF from microglia had no impact on KCC2 expression. ***B***, Lumbar spinal cord sections from animals expressing normal CSF1 (top) and with CSF1 removed from motoneurons (bottom) 14 d after ligation. *Chat^IREScre/+:^ : csf1^f/f^* animals (bottom image) exhibit normal microgliosis in the dorsal horn but have the microglial reaction to injury greatly attenuated in the ventral horn compared to *Chat^I+/+:^ : csf1^f/f^*(top image). Scale bars = 200 μm. ***C***, KCC2 immunofluorescence 14 d after sciatic ligation in *csf1^f/f^* animals. Preventing microgliosis in the ventral horn had no impact on KCC2 within intact or injured motoneurons (Table 5). Error bars = SEM; **p ≤ 0.01, ***p ≤ 0.001.

It is possible that microglia are responsible for the induction of KCC2 loss, while connection with the muscle is required for KCC2 restoration. After PNI, BDNF is upregulated in microglia (Trang et al., 2009), and a large number of studies have reported on the regulation of spinal KCC2 after nerve injuries by microglia-derived BDNF (Coull et al., 2005; Trang et al., 2009; Zhou et al., 2011, 2014; Zhang et al., 2012; Ferrini and De Koninck, 2013; Gomes et al., 2013). To examine whether microglia-BDNF is involved, we used *Cx3cr1^CreERT^*^/+:^ *: bdnf ^f/f^* animals in which *bdnf* is removed specifically from microglia by cre recombination after tamoxifen treatment. In *Cx3cr1^CreERT^*^/+^ animals, one *Cx3cr1* allele is inactivated; therefore, we also included data shown above from WT animals for comparison. All animals (*n* = 5 WT, 6 *Cx3cr1^CreERT2^*) were analyzed 14 d postinjury and compared to sham operated animals of the same genotype. There were significant differences according only to injury, and not genotype or their interaction (Table 5). Deletion of BDNF from microglia or heterozygous inactivation of the *Cx3cr1* gene have no effect on the downregulation of KCC2 after axotomy (Fig. 7*A*).

**Table 5.** Details of statistics for experiments manipulating microglia and motoneuron BDNF production

	Data reference	Data structure	Type of test	Power and statistical significance
A	5A **WT (intact) vs** WT (cut/ligated) Cx3cr1*^CreERT2/+^*::*bdnf* ^ff^ (intact) Cx3cr1*^CreERT2/+^*::*bdnf* ^ff^ (cut/ligated)	Normal distribution	Two-way ANOVA Side; *F*_(1,19)_ = 37.293; *p* < 0.001 Genotype; *F*_(1,19)_ = 0.0715; *p* = 0.792 Interaction; *F*_(1,19)_ = 0.336; *p* = 0.569 *Post hoc* Bonferroni *Post hoc* Bonferroni *Post hoc* Bonferroni	Power = 1.0 Power = 0.05 Power = 0.05 *p* = 0.005 *p* > 0.999 *p* < 0.001
B	5C **Chat^+/+^:: *csf1*^f/f^ (intact) vs** Chat^+/+^:: *csf1* ^f/f^ (cut/ligated) Chat^iREScre/+^:: *csf1* ^f/f^ (intact) Chat^iREScre/+^:: *csf1* ^f/f^ (cut/ligated)	Normal distribution	Two-way ANOVA Side; *F*_(1,19)_ = 25.611; *p* < 0.001 Genotype; *F*_(1,19)_ = 3.665; *p* = 0.074 Interaction; *F*_(1,19)_ = 2.541; *p* = 0.131 *Post hoc* Bonferroni *Post hoc* Bonferroni *Post hoc* Bonferroni	Power = 0.998 Power = 0.322 Power = 0.203 *p* < 0.001 *p* = 0.074 *p* < 0.001
C	6A **Chat^iREScre/+^::*bdnf*^f/+^ (intact) vs** Chat^iREScre/+^:: *bdnf* ^f/+^ (cut/ligated) Chat^iREScre/+^:: *bdnf* ^f/f^ (intact) Chat^iREScre/+^:: *bdnf* ^f/f^ (cut/ligated)	Normal distribution	Two-way ANOVA Side; *F*_(1,17)_ = 22.070; *p* < 0.001 Genotype; *F*_(1,17)_ = 2.417; *p* = 0.142 Interaction; *F*_(1,17)_ = 0.545; *p* = 0.473 *Post hoc* Bonferroni *Post hoc* Bonferroni *Post hoc* Bonferroni	Power = 0.994 Power = 0.188 Power = 0.050 *p* = 0.008 *p* = 0.382 *p* = 0.002
D	6C	Normal distribution	Two-way ANOVA Genotype; *F*_(1,12)_ = 0.443; *p* = 0.518 Cre/YFP; *F*_(1,12)_ = 0.00489; *p* = 0.945 Interaction; *F*_(1,12)_ = 0.0157; *p* = 0.902	Power = 0.05 Power = 0.05 Power = 0.05

Boldface indicates the group against all other comparisons are made in multiple comparisons tests.

To further examine a possible role of microglia independent of BDNF, we attenuated the microglial reaction in the ventral horn by removing the CSF1 gene (*csf1*) from motoneurons using *csf1* floxed alleles and a Chat-iRES-Cre mouse line expressing cre in ChAT-expressing neurons (including all motoneurons). This manipulation was previously shown to prevent ventral horn microgliosis while preserving dorsal horn microglia activation after sciatic nerve injury (Rotterman et al., 2019), and we replicated this effect (Fig. 7*B*). As in WT animals, *Chat ^iREScre/+^*: : *csf1^f/f^* animals and *Chat ^+/+^*: : *csf ^f/f^* (*n* = 5 per group) expressed high levels of KCC2-IR on the cell body of motoneurons contralateral to the injury, while motoneurons axotomized after sciatic nerve cut and ligation were similarly depleted in animals of both genotypes (Table 5). Thus, attenuating the microglia reaction in the ventral horn after nerve injury did not alter motoneuron KCC2 downregulation after axotomy (Fig. 7*C*). We used animals of both sexes (*n* = 2 females, *n* = 3 males per group), and both similarly lose KCC2 with or without ventral horn microglia activation. In conclusion, mechanisms that deplete KCC2 on spinal cord motoneurons following axotomy are independent of microglia in both sexes. This is different from mechanisms inducing KCC2 depletion in dorsal horn neurons following nerve injuries, which involve sex-dependent microglia activation (Mapplebeck et al., 2017).

### KCC2 depletion is independent of motoneuron-BDNF or TrkB activation

**Figure 8.**
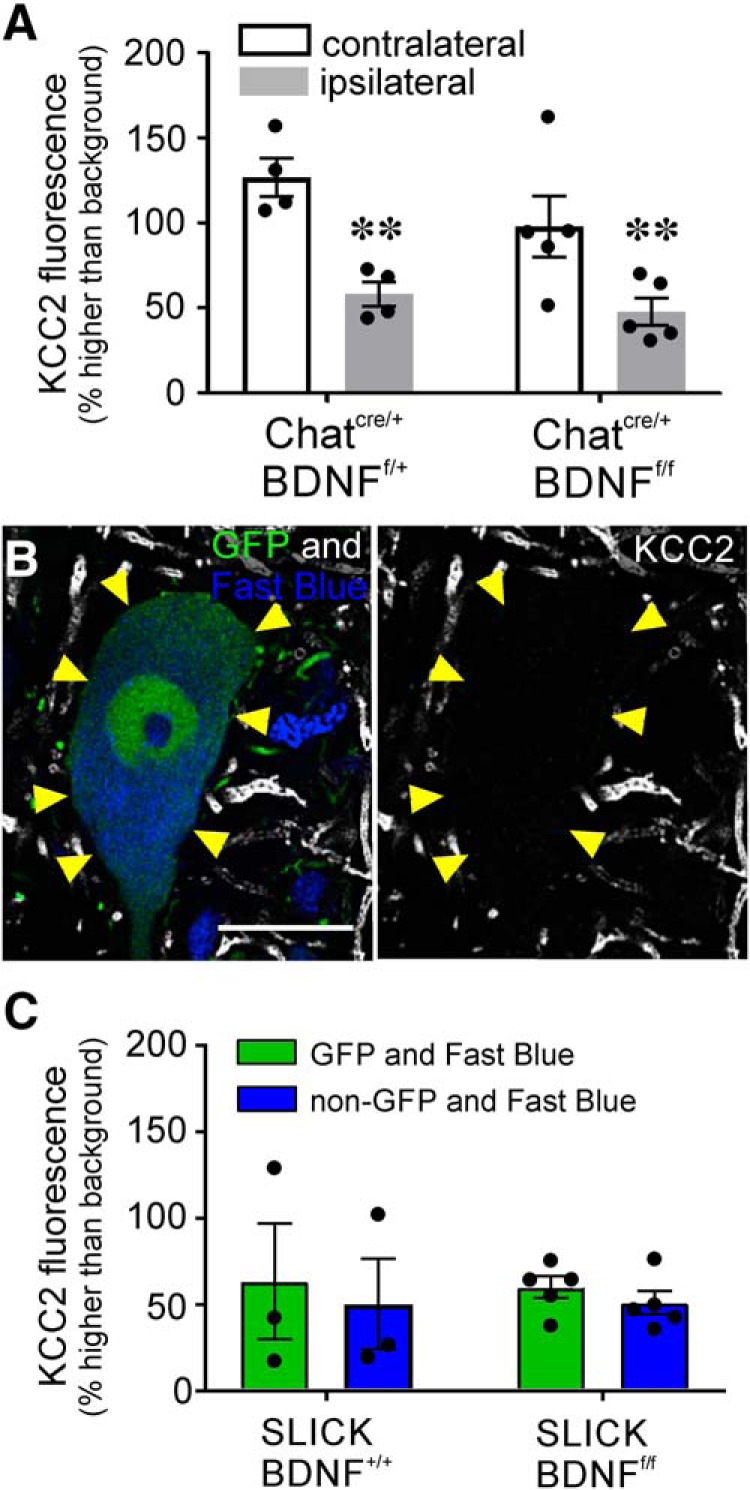
Knock-out of motoneuron BDNF does not impact KCC2 downregulation. ***A***, KCC2-IR in *Chat^IREScre/+:^ : bndf^f/f^* motoneurons 14 d after sciatic cut/ligation. Removal of BDNF from motoneurons throughout development had no impact on KCC2-IR in motoneurons ipsilateral or contralateral to injury. The same baseline levels and depletion were observed regardless of genotype. (Table 5; **p ≤ 0.01). ***B***, SLICK: BDNF^f/f^ LG motoneurons following tamoxifen treatment, 14 d after sciatic nerve cut/ligation. YFP+ neurons express cre and have *bdnf* excised with tamoxifen treatment. KCC2 is extensively removed from the somatic membrane in response to injury even after BDNF KO in adulthood. ***C***, Quantification of KCC2 downregulation on ligated cre+ (YFP+) and cre– (YFP–) motoneurons after tamoxifen treatment and bilateral sciatic ligation (*n* = 4.23 ± 2.75 YFP+ and 4.22 ± 3.35 YFP– motoneurons/animal). There is no difference between cre+ (green) and cre– (blue) motoneuron loss of KCC2 regardless of BDNF KO (Table 5). Error bars = SEM. Removing motoneuron BDNF production had no impact on KCC2 expression or downregulation following peripheral axotomy.

Like microglia, axotomized motoneurons also increase BDNF expression after nerve injuries (Kobayashi et al., 1996; Al-Majed et al., 2000a, 2004). To determine whether autocrine BDNF signaling may induce KCC2 downregulation in axotomized motoneurons, we used *Chat ^iRES-Cre/+:^ : bdnf ^f/f^* mice to knock out *bdnf* from all cholinergic neurons. Removal of BDNF from motoneurons did not impact baseline KCC2 levels or KCC2 decrease after axotomy (Fig. 8*A*). We compared *Chat ^iRES-Cre/+^ : bdnf ^f/+^*(*n* = 4) and *Chat ^iRES-Cre/+^ : bdnf ^f/f^* animals (*n* = 4) and found no significant difference in KCC2-IR levels between uninjured motoneurons and similar statistically significant reductions in injured motoneurons in animals of both genotypes (Table 5).

In these experiments, all cholinergic neurons lacked BDNF throughout development, raising the possibility of compensatory mechanisms. To circumvent this potential pitfall, we analyzed a cohort of animals using SLICK mice. We used specifically the SLICK-A line that we have used previously to delete *bdnf* in a cell-specific manner (Krakowiak et al., 2015). In these animals, YFP and CreER^T2^ are constitutively expressed under the control of the *thy1* promoter in sparse populations of neurons, including some motoneurons. CreER^T2^ is activated in the presence of tamoxifen, which we administered in adulthood, thereby eliminating developmental confounds. This approach has the added advantage of allowing internal controls since only YFP+ cells have the capacity of cre recombination, leaving YFP– populations in the same animals unaffected. We crossed these animals with *bdnf^f/f^* animals and retrogradely labeled LG motoneurons with FB. Thus, all FB+ cells are axotomized LG motoneurons, but only YFP+ and FB+ motoneurons would have undergone cre recombination removing BDNF expression. Due to the sparse YFP/cre expression of motoneurons, 10 YFP/cre+ neurons per animal were not feasible even after doing bilateral injections and axotomy to increase the number of FB/YFP/cre-expressing motoneurons. Membrane KCC2-IR was measured in 2–10 FB/YFP+ motoneurons per animal (4.23 ± 2.75 average neurons/animal ± SD) to obtain each animal average and compared to similar numbers of FB/non-YFP (cre–) neurons (*n* = 2–10 neurons, average 4.22 ± 3.35). We compared SLICK: : *bdnf^+/+^* animals (*n* = 3 animals) with SLICK: : *bdnf^f/f^* animals (*n* = 5 animals) all treated with tamoxifen before FB retrograde labeling and bilateral sciatic nerve cut and ligation. There was no significant difference in KCC2 levels between injured FB+/YFP+ and FB+/YFP– motoneurons (with or without cre, respectively) in either genotype 14 d after injury (Fig. 8*B*,*C*; Table 5). Removing BDNF from adult motoneurons did not prevent KCC2 downregulation, similar to the lack of effects of removing BDNF from all motoneurons throughout development.

### KCC2 depletion is independent of TrkB activation

Removing BDNF from either of the two main sources in the spinal cord after injury did not have any effects on KCC2 regulation, but it is still possible that another source could compensate for motoneuron or microglial BDNF. Moreover, removal of BDNF could also be compensated for by upregulation of neurotrophin-4/5, another ligand of TrkB. It has also been shown that ligand-independent autophosphorylation of TrkB receptors can occur (Zaccaro et al., 2001). We therefore blocked the TrkB receptor directly with either the competitive TrkB antagonist ANA-12 (Cazorla et al., 2011) or by using the F616A mutant pharmacogenetic system (Chen et al., 2005).

**Figure 9.**
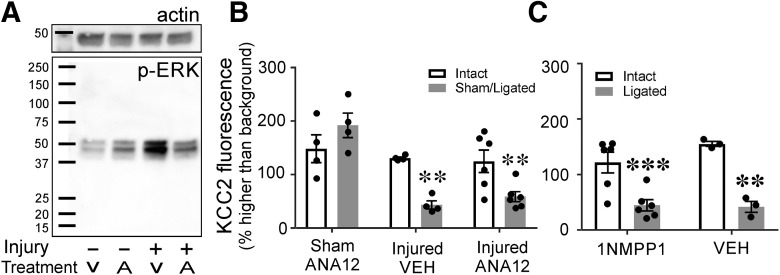
Systemic blockade of TrkB does not attenuate KCC2 downregulation. ***A***, Western blotting of pERK (bottom) and B-actin (top) from lumbar tissue 7 d after bilateral sciatic ligation or sham surgery. Animals were exposed to ANA-12 (a partial TrkB antagonist) or vehicle continuously from time of surgery to killing. In sham animals exposed to ANA-12, there is a slight increase in pERK. However, there was a much larger amount of pERK in vehicle-treated animals that underwent sciatic cut/ligation; this was not seen in injured animals treated with ANA-12. Thus, ANA-12 prevented the normal increase in TrkB signaling that typically occurs after injury. ***B***, KCC2-IR on LG motoneurons of WT animals treated with ANA-12 or vehicle through mini osmotic pumps 14 d after unilateral sciatic sham surgery or cut/ligation. Neither exposure to ANA-12 nor sham surgery altered KCC2 levels in intact motoneurons, and motoneurons with their axons cut and ligated had the same depletion of KCC2 regardless of exposure to ANA-12 (Table 6). ***C***, KCC2-IR on motoneurons of F616A animals 14 d after peripheral cut/ligation. Animals were given 1NMPP1 or vehicle before surgery, through time of killing. F616A animals have a mutated TrkB receptor such that when exposed to 1NMPP1 cannot autophosphorylate or signal. There was no difference in KCC2 presence or loss regardless of treatment (Table 6). Preventing TrkB signaling did not alter KCC2 protein expression or loss after injury (**p ≤ 0.01, ***p ≤ 0.001). Error bars = SEM.

In ANA-12 experiments, mini-osmotic pumps were used to continuously deliver the drug or vehicle intraperitoneally into WT animals, starting at the time of injury and until euthanasia (14 d). Systemic ANA-12 delivered via intraperitoneal injections has been shown to cross the blood brain barrier and effectively inhibit TrkB 4 h postinjection (Cazorla et al., 2011). The concentration of ANA-12 in the pump was adjusted such that we delivered 1 ng/h into the animal. ANA-12 actions were confirmed with Western blotting by probing for pERK, a target of TrkB activation and kinase activity (Wilkerson and Mitchell, 2009). In animals treated with vehicle, pERK expression was increased in the spinal cord of injured animals compared to sham-vehicle-treated animals. This change was attenuated with ANA-12 treatment (Fig. 9*A*). There was no difference in pERK signal between sham and cut ligated animals treated with ANA-12.

To assess any effects of ANA-12 treatment on KCC2 downregulation, we implemented the same quantitative analyses used before on the cell bodies of bilaterally labeled LG motoneurons 14 d following unilateral sciatic nerve cut/ligation. Thus, we compared KCC2 on FB LG motoneurons ipsilateral and contralateral to the injury. In addition, one cohort of animals (*n* = 4) was treated with ANA-12 but underwent a sham injury in one side. In sham control animals, no significant differences in KCC2 membrane immunofluorescence were found on the sham side compared to the contralateral side, and the levels of KCC2-IR was comparable to all control motoneurons in previous experiments. This suggests that basal expression of KCC2 was not affected by ANA-12 treatment. In injured animals, we found similar reductions of KCC2 on injured motoneurons in animals treated with either vehicle (*n* = 4) or with ANA-12 (*n* = 6) compared to control motoneurons (Fig. 9*B*). There were no significant differences in sham injured and intact motoneurons or reductions in KCC2 levels between injured neurons treated with ANA-12 or vehicle (Table 6). In conclusion, ANA-12 treatment did not prevent KCC2 downregulation in axotomized motoneurons.

**Table 6.** Details of statistics for experiments blocking TrkB signaling

	Data reference	Data structure	Type of test	Power and statistical significance
A	7B **Intact ANA-12 vs** sham ANA-12 Intact VEH Injured VEH Intact ANA-12 Injured ANA-12	Normal distribution, unequal variance	Two-way ANOVA Side; *F*_(1,27)_ = 6.474; *p* = 0.018 Treatment; F_(2,27)_ = 13.095; *p* < 0.001 Interaction; *F*_(2,27)_ = 7.289; *p* = 0.004 *Post hoc* Bonferroni *Post hoc* Bonferroni *Post hoc* Bonferroni *Post hoc* Bonferroni *Post hoc* Bonferroni	Power = 0.608 Power = 0.993 Power = 0.860 *p* = 0.576 *p* > 0.999 *p* = 0.003 *p* > 0.999 *p* = 0.006
B	7C **Intact VEH vs** injured VEH Intact 1NMPP1 Injured 1NMPP1	Normal distribution	Two-way ANOVA Side; *F*_(1,17)_ = 35.783; *p* < 0.001 Treatment; *F*_(1,17)_ = 0.935; *p* = 0.350 Interaction; *F*_(1,17)_ = 1.325; *p* = 0.269 *Post hoc* Bonferroni *Post hoc* Bonferroni *Post hoc* Bonferroni	Power = 1.00 Power = 0.05 Power = 0.079 *p* = 0.002 *p* = 0.470 *p* < 0.001

Boldface indicates the group against all other comparisons are made in multiple comparisons tests.

ANA-12 is a partial competitive antagonist of TrkB (Chen et al., 2015) and therefore we cannot assume block of ligand binding to be complete. To obtain further proof of a lack of a role for TrkB in KCC2 downregulation, we blocked TrkB kinase activity (and thus signaling) using F616A mutant mice. F616A animals express a TrkB mutation with a single base substitution that makes it sensitive to the kinase inhibitor 1NMPP1 (Chen et al., 2005). 1NMPP1 was administered in the drinking water using established protocols that prevent TrkB downstream signaling (Chen et al., 2005; Wang et al., 2009; Rantamäk et al., 2011). In agreement with ANA-12 experiments, in F616A animals treated with normal drinking water (*n* = 3) and 1NMPP1 (*n* = 6) normal levels of KCC2 were found on uninjured FB LG motoneurons. A downregulation of KCC2 was also found after injury in FB LG motoneurons regardless of treatment (Table 6; Fig. 9*C*). Taken together, the results in ANA-12-treated animals and F616A animals allow us to conclude that KCC2 downregulation in axotomized motoneurons is independent of TrkB signaling.

## Discussion

In this study, we demonstrate that KCC2-IR is significantly decreased on the cell membranes of spinal motoneuron somata and dendritic processes following peripheral axotomy. KCC2 disappearance begins with a downregulation of KCC2 mRNA in the days immediately after injury, followed by a slower disappearance of protein from the membrane lasting weeks. We also found that KCC2 is restored only after successful axon regeneration and muscle reinnervation, raising the possibility that peripheral, rather than central, signals may regulate KCC2 expression. Furthermore, KCC2 regulation on peripherally axotomized motoneurons occurs independent of microglia and BDNF/TrkB signaling. This is different from the canonical mechanism for KCC2 downregulation that has been established in dorsal horn neurons. Instead, KCC2 downregulation on spinal motoneurons is more similar in time course and extent to what has been previously reported in brainstem motor nuclei following peripheral axotomy.

### KCC2 is preferentially preserved in distal dendrites

Retention of KCC2 in the distal dendrites of spinal motoneurons, despite strong reductions in KCC2 mRNA, suggests differences in KCC2 stability in different dendrite compartments. Dendrites also lose most synapses on proximal, rather than distal, compartments after axotomy (Brännström and Kellerth, 1998; Rotterman et al., 2014). Interestingly, electrophysiological studies have also shown that glycinergic (strychnine-sensitive) synapses, found mostly on the somata (Takata and Ogata, 1980) sustained larger decreases in efficacy than more distally located GABAergic (picrotoxin-sensitive) synapses (Takata and Ogata, 1980; Takata and Nagahama, 1983). It is worth noting that in some central neurons, distal dendrites are enriched with the KCC2a isoform (Markkanen et al., 2014) and that KCC2 in dendrites is frequently associated with the maintenance of structural properties at excitatory synapses and less with a chloride transport function (Gulyás et al., 2001; Blaesse and Schmidt, 2015). Recent data from using isotype specific antibodies (P. Calvo and F. J. Alvarez, unpublished observations) shows that KCC2b is uniquely expressed on the somatic and proximal dendrite membrane of spinal motoneurons. Possible differences in KCC2 isoforms, as well as anchoring, membrane stabilization, and function in distinct dendritic compartments are all possibilities for explaining the differential loss of KCC2 in motoneuron soma and dendrites that deserve further study.

It should also be noted that, for the first time, we have reported trafficking of *kcc2* mRNA into dendritic compartments of motoneurons. KCC2 transcription of *kcc2* mRNA within the dendrites is one alternative explanation for the differential regulation of dendritic KCC2. Our Nissl-stained sections only permitted analyses of proximal dendrites as they emerge from the cell body. Future studies should be performed combining RNA-Scope *kcc2* mRNA detection and dendritic labeling with AAV1 transfection to localize *kcc2* mRNA throughout the dendritic arbor. The high sensitivity and specificity of the RNA-Scope technique would allow detection of small quantities of mRNAs in dendritic compartments. Nonetheless, we believe it is unlikely that there is enough preservation of *kcc2* mRNAs in distal dendrites to maintain KCC2 protein; the scattered dots of mRNA signal in the neuropil adjacent to the axotomized motoneurons also disappeared after injury. Therefore, the best explanation at present is that KCC2 in the very distal dendrites has a very low turnover rate, explaining their preservation at the time point of our analyses. In this case, we would expect chronic axotomy at longer time points would result in the eventual loss of this KCC2 as well.

It is also possible KCC2 in distal dendrites is serving a different purpose. Independent of its role in ion transport, KCC2 has been shown to stabilize excitatory synapses and be important in dendritic spine development by regulating actin phosphorylation and stabilization (Li et al., 2007; Llano et al., 2015). KCC2-deficient mice have fewer functional glutamatergic synapses and excessively long dendritic protrusions that cause an increase in the total dendrite length of pyramidal cells (Li et al., 2007). The regions where we see KCC2 preservation on motoneurons (the last 75–100 μm of dendrite) typically have very low synaptic densities (Alvarez et al., 1997), making synapse stabilization an unlikely explanation for KCC2 retention there. However, these most distal regions of the dendrites undergo many changes after long-term axotomy without regeneration, including becoming more highly branched, increasing in diameter, and becoming axon-like in both structure and molecular composition (Rose and Odlozinski, 1998; Rose et al., 2001; MacDermid et al., 2002). At the time points we observed, no drastic changes in distal dendrite morphology were evident, but it is possible that the high density of KCC2, if lost long-term, is contributing to the eventual morphologic changes observed with longer-term axotomy (Rose and Odlozinski, 1998).

### KCC2 regulation in axotomized motoneurons

The first study of KCC2 regulation on motoneurons following PNI was conducted in the dorsal motor nucleus of the vagus (DMV), where KCC2 mRNA was diminished by 3 d after vagal nerve axotomy in young rats (Nabekura et al., 2002). DMV neurons innervate postganglionic parasympathetic neurons rather than skeletal muscle, but these findings were later recapitulated in facial (Toyoda et al., 2003; Kim et al., 2018) and hypoglossal (Tatetsu et al., 2012) motoneurons following transection of the respective peripheral nerves. Several of these studies speculated on the eventual return of KCC2 mRNA (Toyoda et al., 2003) and protein (Tatetsu et al., 2012; Kim et al., 2018), but none analyzed this directly or whether it was dependent on successful target reinnervation. Our study established that KCC2 recovery in axotomized motoneurons is correlated with successful axon regeneration and more specifically with re-innervation of NMJs, this mechanism could be shared by both brainstem and spinal motoneurons.

While brainstem and spinal motoneurons both innervate muscle, it should be noted that the circuitry surrounding them is vastly different and responses to injury may not always be equivalent across motoneuron populations. Whereas facial (Vaughan, 1994; Kim et al., 2018), like spinal motoneurons (Alvarez et al., 2011; Rotterman et al., 2019) have small decreases in gephyrin+ (GABA/glycinergic) synapses following peripheral axotomy, hypoglossal motoneurons do not lose inhibitory synapses (Sumner, 1975; Tatetsu et al., 2012). Interestingly, inhibitory synapses on facial motoneurons increased in size and GABA content following axotomy (Vaughan, 1994). While this has not been investigated on spinal motoneurons following PNI, it is possible that they also shift their inhibitory synapse phenotype or strength following injury. Many inhibitory synapses in the spinal cord are mixed GABA/glycine synapses, and they shift from predominantly GABAergic to predominantly glycinergic during postnatal development (Gao et al., 2001; González-Forero and Alvarez, 2005). Thus, adult spinal motoneurons predominantly receive inhibition from glycinergic synapses (Alvarez et al., 1997) but could revert to GABAergic mechanisms after axotomy. Postsynaptic GABA_A_ synaptic currents have much slower decay times than glycinergic synapses (Alvarez, 2017), allowing for longer depolarizations in the context of diminished KCC2 expression and high internal chloride. During development, while KCC2 levels are low, high internal chloride and long-lasting GABAergic depolarizations drive neurite outgrowth (Sernagor et al., 2010). Thus, in contrast to dorsal horn neurons after PNI or motoneurons following SCI (which highlight disinhibition after KCC2 loss), KCC2 changes on axotomized brainstem motoneurons seems to induce a state in which GABAergic transmission is the direct driver of activity. Correspondingly, loss of KCC2 was coupled with increased calcium levels and firing in response to GABA exposure in slice preparations of axotomized DMV motoneurons (Nabekura et al., 2002) and with spontaneous calcium oscillations in axotomized facial motoneurons that were driven by depolarizations dependent on activation of GABA_A_ and NMDA receptors (Toyoda et al., 2003).

### KCC2 regulation after PNI differs between motoneurons and dorsal horn interneurons

Like motoneurons, dorsal horn spinal neurons (more specifically nociceptive Lamina I neurons) decrease KCC2 following nerve injuries, resulting in disinhibition and behavioral hyperalgesia (Coull et al., 2003, 2005). Unlike motoneurons, dorsal horn neurons are fully contained within the CNS and are not directly injured or axotomized after PNI. Instead, signals from injured sensory afferents trigger the activation of microglia in the dorsal horn and subsequent release of BDNF that depletes KCC2 in neighboring neurons, at least in males (Tsuda et al., 2003; Coull et al., 2005; Trang et al., 2009, 2012; Beggs et al., 2012; Sorge et al., 2015). Dorsal horn neurons in female mice also decrease KCC2 but through a different mechanism than males that is not yet well elucidated (Mapplebeck et al., 2018). Independent of sex, the changes in KCC2 occur quickly following injury, most typically a constriction of the nerve, and are likely due to changes in KCC2 phosphorylation and membrane trafficking. In axotomized motoneurons, however, KCC2 is downregulated at the transcriptional level and it takes two to three weeks for complete loss of the protein from the membrane, likely because of slow protein turnover.

Another major distinction between spinal motoneurons after PNI and mechanisms described in other models is a differential dependence on microglia and BDNF. BDNF-TrkB can regulate KCC2 membrane content by post-translational mechanisms, altering KCC2 phosphorylation, or directly by regulating KCC2 gene expression (for review, see Lee-Hotta et al., 2019). The cell bodies of axotomized motoneurons are quickly surrounded by a layer of activated microglia (Aldskogius, 2011) that, similar to dorsal horn microglia, could be involved in regulating KCC2 expression through BDNF release or other mechanisms. However, our results negate this hypothesis, demonstrating a lack of involvement of ventral microglia on the downregulation of KCC2 in axotomized motoneurons. Microglia activation phenotypes in the dorsal and ventral horn after PNI are not necessarily the same, and differences might further increase depending on the type of injury. Although activation of microglia in the dorsal horn is also dependent on CSF1, this time released from affected primary afferents (Guan et al., 2016), the release of BDNF from activated dorsal horn microglia was shown to specifically depend on microglia P2X4 receptors activated by ATP released from the central terminals of overactive sensory afferents (Beggs et al., 2012). Ventral horn microglia also upregulate several classes of purinergic receptors after nerve injury (Kobayashi et al., 2012), but their exact roles have not yet been investigated. Although ventral horn microgliosis is also dependent on CSF1 released by axotomized motoneurons (Rotterman et al., 2019), some features of the microglia reaction to nerve injury differ between the dorsal and ventral horn (Rotterman et al., 2019). Another significant difference is the type of nerve injury leading to activation of microglia. Dorsal horn studies are focused on a model of partial ligation of the sciatic nerve known to cause a well-characterized hyperalgesia (Bennett and Xie, 1988), while we studied the consequences of full transection of the nerve. One study comparing the phenotype of dorsal horn microglia in these two types of injuries found many significant differences (Hu et al., 2007). In conclusion, the response of microglia in the ventral horn is not involved in the downregulation of KCC2 from motoneurons after nerve transections and is thus different from the well-established role of dorsal horn microglia, at least in males, after hyperalgesia-inducing nerve injuries.

### KCC2 downregulation is independent of TrkB activation

BDNF is transiently upregulated in motoneurons following axotomy (Kobayashi et al., 1996; Al-Majed et al., 2000a, 2004), and the release of BDNF is key in treatments that enhance motor axon regeneration and functional recovery following injury (Al-Majed et al., 2000a,b; English et al., 2007, 2013; Wilhelm et al., 2012; Krakowiak et al., 2015). BDNF has also been implicated in the reduction of KCC2 inducing spasticity after SCI (Boulenguez et al., 2010). However, based on the results of our experiments showing little effect on the loss of KCC2 after axotomy after knocking out the *bdnf* gene in motoneurons, either through development or selectively in adults before injury, along with blocking TrkB signaling with ANA-12 or in the F616A mice, we conclude that BDNF/TrkB mechanisms are not involved in the regulation of KCC2 expression in axotomized motoneurons after PNI. Intriguingly, we found evidence for a possible dependence on target innervation. It is thus possible that peripherally derived signals directly modulate KCC2 gene expression in axotomized motoneurons independent of BDNF and TrkB activation.

### Comparison of KCC2 downregulation in motoneurons after nerve injury or SCI

Following SCI, motoneurons below the level of injury also partially lose KCC2 (Boulenguez et al., 2010). These motoneurons are not directly injured during SCI and their axons remain intact in the peripheral nerve. A post-translational mechanism has been proposed to explain this effect, indicating that KCC2 downregulation was dependent on the loss of descending serotonergic inputs and 5HT2A receptor activity (Bos et al., 2013). The downregulation of KCC2, as measured in just the cell body, was estimated to be around 20% (but may be better preserved on the dendrites). This lead to a small but significant increase in the reversal potential of chloride of only 5 mV in adult rats (Murray et al., 2011) that was sufficient to generate significant disinhibition and spasticity. After SCI, motoneuron excitability and spasticity can be rectified with enhancers of KCC2 activity (Sánchez-Brualla et al., 2018), suggesting that enough KCC2 remains on the surface of the motoneurons to rescue KCC2 function and the animal from spasticity. In contrast, after axotomy of adult facial motoneurons, E_GABA_ depolarizes by almost 19 mV (Toyoda et al., 2003). In facial motoneurons and spinal motoneurons, almost no KCC2 mRNA or protein is present two weeks after injury, and we have shown that the loss includes substantial regions of the dendritic arbor. Thus, while downregulation of KCC2 in motoneurons after SCI leads to a partially disinhibited state, the more complete downregulation we describe here might lead to GABA/glycine synapses reversals climbing above action potential thresholds and becoming significant drivers of activity in regenerating motoneurons. Given the drastic change in KCC2 on axotomized motoneurons and the close link of this loss with regenerative state, it is possible that KCC2 reductions may be tied to motoneuron regenerative mechanisms. Activity-dependent treatments are known to enhance regeneration (Al-Majed et al., 2000a,b; English et al., 2007, 2009; Udina et al., 2011) and this enhancement could be complemented by the many changes in the physiology of regenerating motoneurons that increase their excitability: increased input resistance, decreased rheobase, and changes in action potential dynamics (Kuno et al., 1974; Huizar et al., 1977; Gustafsson, 1979; Gustafsson and Pinter, 1984; Gonzalez-Forero et al., 2007). In this context KCC2 depletion resulting in enhanced “excitatory” drive from GABA/glycine synapses may further promote motoneuron firing and calcium entry, contributing to regeneration. This is a hypothesis that will need to be fully investigated in the future. It is however important to consider at present the possibility that while KCC2 depletion in the adult spinal cord can be maladaptive in some conditions (SCI spasticity, hyperalgesia), it might be beneficial in other situations (motoneuron regeneration after PNI).

In summary, KCC2 downregulation in spinal interneurons versus motoneurons after PNI and in motoneurons in SCI versus PNI differ in mechanism, completeness, and functional role. This should be taken into account when assessing the potential impact of KCC2-enhancing manipulations after different kinds of injuries.

## Synthesis

Reviewing Editor: Jeffery Twiss, University of South Carolina Decisions are customarily a result of the Reviewing Editor and the peer reviewers coming together and discussing their recommendations until a consensus is reached. When revisions are invited, a fact-based synthesis statement explaining their decision and outlining what is needed to prepare a revision will be listed below. The following reviewer(s) agreed to reveal their identity: Zhigang He, Leif Havton.

The authors examined the mechanisms of KCC2 down-regulation in axotomized spinal motor neurons with and without reinnervation of muscle. They first established a system to measure KCC2 expression throughout somatodendritic membranes. Next, they used genetic approaches to demonstrate that microglia, BDNF, and TrKB are not responsible for the KCC2 down-regulation caused by sciatic transection; instead, KCC2 is dependent on neuromuscular innervation. Overall, the reviewers felt that the study is well done and includes a comprehensive set of experiments addressing fundamental questions related to factors contributing to the downregulation of KCC2 expression in motor neurons. However, reviewers and the reviewing editor agreed that there are a few issues to be addressed before further considering this work for publication. Major considerations are on the interpretation of the results for KCC2 regulation mechanism, neuron types expressing KCC2 (and not expressing), and KCC2 internalization vs. protein loss:

1. While the majority of KCC2 analysis was conducted at 14 days after injury and the authors claimed transcription as a key regulatory step, it is unknown if the same mechanism applies to KCC2 down-regulation at early post-injury time points. In Fig. 4, the authors reported a modest reduction KCC2 signal. It would be important to assess whether such signals are associated with the plasma membrane and/or dendrites and the KCC2 mRNA levels. As numerous reports have documented KCC2 internalization in injured neurons, such assessments are needed to assess the contribution of these different mechanisms to KCC2 reduction.

2. In Figure 3, bright field images show labeling for KCC2 mRNA of both large and small neurons in the motor pool with loss of labeling for KCC2 mRNA in the large neurons after injury. The large neurons are interpreted as being motor neurons and the small neurons are interpreted as being interneurons. If these images were captured from Rexed's lamina IX, the large cells are likely alpha-motor neurons, since most interneurons in the ventral horn would be expected in Rexed's lamina VII and VIII. Given that ventral horn motor neurons normally show bimodal size distributions with the larger neurons being alpha-motor neurons and the smaller neurons being gamma-motor neurons, it is plausible that the smaller cells in the images are gamma-motor neurons rather than interneurons. Do the authors have any other information on the nature or characteristics of the smaller cells to address this issue? If there is indeed a consistent difference in labeling between the small and large neurons in lamina IX after the axotomy, it raises the possibility that gamma- and alpha-motor neurons may respond differently to injury with regards to KCC2 mRNA expression.

3. The hypothesis in the article is to test if BDNF/TrKB activation induces KCC2 down-regulation. Although the authors have demonstrated that pharmaceutical block of TrKB receptor does not influence KCC2 expression (Figure 7), this approach lacks specificity as there is no way to know the motor neurons' TrKB activity status after ANA12 treatment. A more definitive experiment is to delete TrKB receptor in motor neurons (by using the SLICK mice applied in the paper), and then to test KCC2 expression level. This would provide direct evidence of any correlation between motor neuron BDNF/TrKB activation and KCC2 expression. If BDNF activation has an effect in KCC2 expression, then it would be necessary to test different secretion source of BDNF to see which one is responsible for such down-regulation, as the authors did in Figure 5-6.

4. In the series of animals with reinnervation of muscle being allowed, was there any additional evidence for successful reinnervation - for instance, muscle weight measurements?

5. The analysis of the differential depletion of KCC2 immunoreactivity along the dendritic tree with proximal dendrites shows depletion after the axotomy, whereas the distal dendrites in the same neurons are more likely to be unaffected is interesting. A prior study of axotomy of motor neurons have also shown that the synapse elimination is most pronounced in the proximal parts of the dendritic tree with relative synapse preservation in the distal arbors (Brannstrom and Kellerth, Exp Brain Res, 1998). Some expansion on discussion of this association is suggested.

Minor concerns:

1. Figure 1D, it is not possible to see the shading that indicates male vs. female.

2. In Figure 2, how were the axons identified as distinct from a non-branching dendritic segment in the 50 um thick spinal cord section?

3. Figure 3, which segmental level is the dark field image from?

4. KCC2 expression seems to return to normal levels after neuromuscular innervation, which is an interesting observation, but it raises some questions. The authors have not shown that the KCC2 protein re-appears at the membrane after muscular innervation, which is important data not included here. For KCC2 membrane fluorescence returning to intact levels after LG muscular innervation occurred, was this only for LG muscle or is it the same for other muscles?

5. It would be interesting to know what the mechanism to promote KCC2 re-appearance on the membrane after muscle innervation. Does re-innervation reduce KCC2 degradation or increase KCC2 protein synthesis? Testing how KCC2 mRNA expression levels after re-innervation could help to address this (or at least help point to mechanism).

6. The authors should clarify that the dendritic tree visualized in a single slices does not include the entire dendritic arbors for the motor neurons.

7. Suggest addition of information in Methods with regards to which software product and version that was used for the statistical analyses.
